# Structural basis of neuropeptide Y signaling through Y_1_ and Y_2_ receptors

**DOI:** 10.1002/mco2.565

**Published:** 2024-06-15

**Authors:** Siyuan Shen, Yue Deng, Chenglong Shen, Haidi Chen, Lin Cheng, Chao Wu, Chang Zhao, Zhiqian Yang, Hanlin Hou, Kexin Wang, Zhenhua Shao, Cheng Deng, Feng Ye, Wei Yan

**Affiliations:** ^1^ Division of Nephrology and Kidney Research Institute State Key Laboratory of Biotherapy West China Hospital Sichuan University Chengdu Sichuan China; ^2^ Frontiers Medical Center Tianfu Jincheng Laboratory Chengdu China; ^3^ Institutes for Systems Genetics Frontiers Science Centre for Disease‑Related Molecular Network West China Hospital Sichuan University Chengdu Sichuan China; ^4^ Department of Otolaryngology Head and Neck Surgery Sichuan Provincial People's Hospital University of Electronic Science and Technology of China Chengdu China; ^5^ Department of Pathology Institute of Clinical Pathology Frontiers Science Center for Disease‐related Molecular Network West China Hospital of Sichuan University Chengdu China

**Keywords:** G protein coupled receptor, ligand selectivity, neuropeptide Y, neuropeptide Y receptors

## Abstract

Neuropeptide Y (NPY), a 36‐amino‐acid peptide, functions as a neurotransmitter in both the central and peripheral nervous systems by activating the NPY receptor subfamily. Notably, NPY analogs display varying selectivity and exert diverse physiological effects through their interactions with this receptor family. [Pro^34^]–NPY and [Leu^31^, Pro^34^]–NPY, mainly acting on Y_1_R, reportedly increases blood pressure and postsynaptically potentiates the effect of other vasoactive substances above all, while N‐terminal cleaved NPY variants in human body primary mediates angiogenesis and neurotransmitter release inhibition through Y_2_R. However, the recognition mechanisms of Y_1_R and Y_2_R with specific agonists remain elusive, thereby hindering subtype receptor‐selective drug development. In this study, we report three cryo‐electron microscopy (cryo‐EM) structures of Gi2‐coupled Y_1_R and Y_2_R in complexes with NPY, as well as Y_1_R bound to a selective agonist [Leu^31^, Pro^34^]–NPY. Combined with cell‐based assays, our study not only reveals the conserved peptide‐binding mode of NPY receptors but also identifies an additional sub‐pocket that confers ligand selectivity. Moreover, our analysis of Y_1_R evolutionary dynamics suggests that this sub‐pocket has undergone functional adaptive evolution across different species. Collectively, our findings shed light on the molecular underpinnings of neuropeptide recognition and receptor activation, and they present a promising avenue for the design of selective drugs targeting the NPY receptor family.

## Graphical abstract

Neuropeptide Y (NPY) serves a critical role in modulating a variety of physiological processes. Gaining comprehensive understanding of structural mechanisms through which Y_1_R and Y_2_R interact with NPY is indispensable for the rational design of selective drugs. We offer detailed molecular maps depicting the binding of NPY peptides to NPY receptor subtypes, thereby shedding light on subtype‐specific interaction patterns.

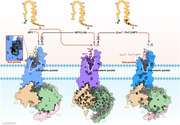

## 1 INTRODUCTION

The neuropeptide Y (NPY) receptor subfamily belongs to class A G‐protein coupled receptor (GPCR) β branch and comprises four receptor subtypes (Y_1_R, Y_2_R, Y_4_R, and Y_5_R), mediating different types of effector proteins signaling pathways, including Gi or Go proteins.^1^, ^2^, ^3^ They can be activated by three structurally related, but functionally diverse endogenous peptides: NPY, peptide YY, and pancreatic polypeptide (PP).^4^, ^5^ All three peptides comprise 36 amino acids each with amidated C‐terminal ends and share highly conserved amino acid sequences.^6^, ^7^ Among them, NPY could specifically activate Y_1_R, Y_2_R, and Y_5_R, and it is broadly distributed in the central and peripheral nervous system, regulating various physiological processes such as food intake, stress response, anxiety, and memory retention.^8^, ^9^

NPY is cleaved into two N‐terminal truncated NPY variants during cellular metabolism: NPY^3−36^ and NPY^2−36^, processed by dipeptidyl peptidase IV and aminopeptidase P (AmP), respectively.^10^, ^11^, ^12^, ^13^ The truncated NPY peptides lose their binding affinity for Y_1_R, but they retain a similar binding affinity with Y_2_R.^14^, ^15^ In addition, the synthesized NPY analog [Leu^31^, Pro^34^]–NPY is reported to be specific agonist at Y_1_R, sharing similar activation potency with NPY for Y_1_R.^16^, ^17^, ^18^, ^19^ Y_1_R and Y_2_R are known to be primarily expressed at the post‐ and presynaptic membranes, respectively.^20^, ^21^, ^22^ [Pro^34^]–NPY and [Leu^31^, Pro^34^]–NPY, when acting on Y_1_R, primarily raise blood pressure and postsynaptically potentiate the effects of other vasoactive substances. In contrast, N‐terminally truncated NPY mostly mediates angiogenesis and inhibits neurotransmitter release through Y_2_R‐mediated processes.^23^, ^24^, ^25^, ^26^, ^27^ These results suggest that Y_1_R and Y_2_R exhibit different ligand recognition mechanisms and could potentially allow for selective drug development for corresponding condition. However, selective agonist development would require the identification of the underlying mechanisms of receptor–peptide ligand interactions. Although structural information of Y_1_R‐ and Y_2_R has been reported,^28^, ^29^, ^30^ the underlying ligand selectivity and receptor activation mechanisms remain unclear. Recently, structural studies on the NPY_1_R have elucidated the molecular mechanisms underlying Y_1_R recognition of NPY. However, the molecular basis for why Y_1_R requires the complete N‐terminus of NPY for optimal activation and why [Leu^31^, Pro^34^]–NPY selectively activate Y_1_R rather than Y_2_R remains unclear. Our study has provided new insights to address these mentioned issues.

In this study, we present three single‐particle cryo‐electron microscopy (cryo‐EM) structures of the Y_1_R/Y_2_R–Gi2 signaling complexes bound to endogenous peptide NPY, as well as Y_1_R–Gi2 in complex with a selective ligand [Leu^31^, Pro^34^]–NPY. Moreover, we performed structural comparison and identified a unique sub‐pocket in Y_1_R that accommodates the N‐terminal NPY residues. Further, the results of our adaptive evolution analysis demonstrated that positive Darwinian selection occurred specifically in Y_1_R from Osteichthyes. In addition, our structural analysis reveals that the residue Pro^34^ substitution in NPY specifically interrupted the extensive NPY–Y_2_R interaction but is more compatible with Y_1_R. Our study elucidates key residues required for NPY peptide recognition and deciphers the plasticity of orthosteric site of NPY receptor subtypes in response to the same ligand stimuli.

## 2 RESULTS

### 2.1 Cryo‐EM structures of Y_1_R/Y_2_R–Gi2 signaling complexes

**FIGURE 1:**
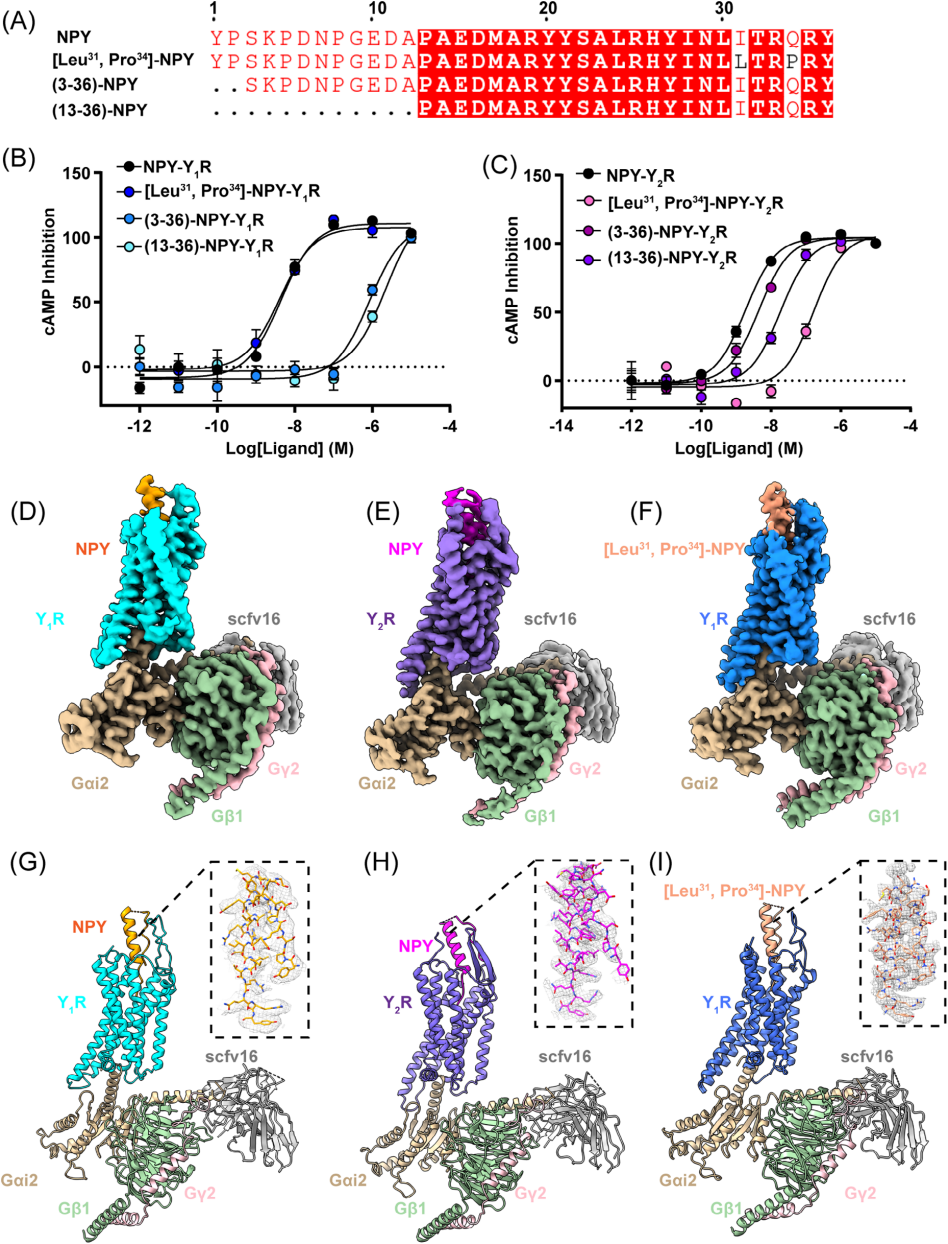
Cryo‐EM structures of NPY–Y_1_R–Gi2, [Leu^31^, Pro^34^]–NPY–Y_1_R–Gi2, NPY–Y_2_R–Gi2 complexes. (A) Sequence alignment of different N‐terminally truncated NPY and [Leu^31^, Pro^34^]–NPY. The alignment was generated by ESPript3 with CLUSTALW (red squares, identical residues). (B) Concentration–response curves of Y_1_R in response to stimulation with NPY, [Leu^31^, Pro^34^]–NPY, NPY^3–36^, and NPY^13–36^, respectively. Values are shown as the mean ± s.e.m. of three experiments (*n* = 3) performed in triplicate. (C) Concentration–response curves of Y_2_R in response to stimulation with NPY, [Leu^31^, Pro^34^]–NPY, NPY^3–36^, and NPY^13–36^, respectively. Values are shown as the mean ± s.e.m. of three experiments (*n* = 3) performed in triplicate. (D) Orthogonal views of the electronic density map of the Y_1_R‐NPY–Gi2–complex. The Y_1_R and NPY are colored cyan and orange, respectively; and Gαi_2_, Gβ, Gγ, and scFv16 are colored tan, dark cyan, pink and silver. (E) Orthogonal views of the electronic density map of the Y_2_R–NPY–Gi2–complex. The Y_2_R and NPY are colored medium slate blue and magenta, respectively; and Gαi, Gβ, Gγ, and scFv16 are colored tan, dark cyan, pink, and silver. (F) Orthogonal views of the electronic density map of the Y_1_R–[Leu^31^, Pro^34^]–NPY–Gi2–complex. The Y_1_R and [Leu^31^, Pro^34^]–NPY are colored medium blue and light salmon, respectively; and Gαi, Gβ, Gγ, and scFv16 are colored tan, dark cyan, pink, and silver. (G ‐I ) Ribbon representation of the Y_1_R–NPY–Gi2, Y_2_R–NPY–Gi2 and Y_1_R–[Leu^31^, Pro^34^]–NPY–Gi2 complexes, colored according to Cryo‐EM maps.

In order to investigate Y_1_R and Y_2_R activation potency in response to NPY variants, including N‐terminally truncated NPY and NPY mutants (Figure 1A), we measured the cAMP inhibition ability of wildtype Y_1_R and Y_2_R elicited by different agonists in HEK293 cells (Figures 1A–C). Our results indicated that deleting the N‐terminal residues (Y^1^ and P^2^) of NPY (NPY^3−36^) and NPY^13−36^ remarkably reduced Y_1_R activation compared to NPY (>50 folds), whereas [Leu^31^, Pro^34^]–NPY analog retained a potency nearly identical to NPY (Figure 1B). In contrast, the NPY^3−36^ variant exhibited slightly reduced Y_2_R activation and [Leu^31^, Pro^34^]–NPY was ∼100‐fold less potent than NPY on Y_2_R activation (Figure 1C). These observations suggest that Y_1_R and Y_2_R display different ligand recognition mechanisms.

To gain a more thorough understanding of how Y_1_R and Y_2_R recognize NPY or NPY mutants, we determined the structures of NPY‐bound Y_1_R, NPY‐bound Y_2_R, and [Leu^31^, Pro^34^]–NPY‐bound Y_1_R in complex with the Gi2 protein using cryo‐EM single‐particle technique (Figures 1D–I and S1 and 2, and Table S1). The final cryo‐EM maps of NPY–Y_1_R–Gi2 (PDB ID:8K6M), NPY–Y_2_R–Gi2 (PDB ID:8K6N), and [Leu^31^, Pro^34^]–NPY–Y_1_R–Gi2 (PDB ID: 8K6O) displayed global nominal resolutions of 3.5, 3.2, and 3.3 Å after refinement, respectively (Figures S1 and 2 and Table S1). The Y_1_R and Y_2_R structure shared a canonical seven‐transmembrane (TM) helical architecture and an intracellular amphipathic helix 8. The seven TM helix domains of both receptors could be visibly distinguished in the cryo‐EM maps (Figure S3), and the high‐resolution density maps allow us to unambiguously build the NPY, [Leu^31^, Pro^34^]–NPY, NPYRs, Gαi_2_, Gβ, Gγ, and scFv16 (Figures 1D–I and S3). However, probably due to the NPY flexibility in the extracellular region, we failed to model the residues 10−15 from NPY and [Leu^31^, Pro^34^]–NPY and the residues 10−14 from NPY in Y_1_R and Y_2_R complex structures, respectively (Figures 1G–I).

By comparing NPY–Y_1_R or NPY–Y_2_R with their inactive states (PDB ID: 5ZBQ and 7VGX, respectively),^31^, ^32^ we noticed that the intracellular end of TM6 in two receptors exhibited an obvious outward shift to accommodate the Gαi_2_ α5 helix and TM7 also adopted an inward displacement similar to other active class A GPCR structures^33^, ^34^, ^35^ (Figures S4A and B). As the NPY–Y_1_R and [Leu^31^, Pro^34^]–NPY–Y_1_R structures share highly similar conformation with a Cα root mean square deviation (RMSD) of 0.569 Å (279–279 atoms), we use the NPY–Y_1_R–Gi2 and NPY–Y_2_R–Gi2 structures to analyze the overall Y_1_R and Y_2_R structures in this section (Figure S4C).

Although Y_1_R and Y_2_R shared only 30% sequence identity, these two receptors activation complex structures resembled a similar conformation with a Cα RMSD of 0.905 Å (213–213 atoms) (Figure S4D). It is noteworthy that the extracellular end of TM1, TM4, and TM5 in Y_1_R exhibited a slight outward movement relative to that in Y_2_R (Figure S4D). The ligand NPY displayed similar binding pattern both in the Y_1_R and Y_2_R structures and folded into a canonical PP‐fold,^36^ the C‐terminal regions (residues 32−36) were inserted into an orthosteric pocket of Y_1_R and Y_2_R, while the N‐terminals (residues 1−10) folded back to form extensive interactions with the NPY α‐helix (residues 15−31) (Figures 1G–H and S5A–C).

### 2.2 A sub‐pocket in Y_1_R determines NPY selectivity

The NPY–Y_1_R with NPY–Y_2_R structural comparison revealed remarkable displacements (∼9 Å) in the NPY N‐terminal part (Figure S5D). In Y_1_R, the NPY N‐terminal end could be found in a cavity composed of the extracellular ends of TM5, TM6, and ECL2. However, the NPY N‐terminal part in Y_2_R extended toward the ECL2 region, forming relatively few contacts with the receptor. In accordance with the NPY pharmacological assay, the structure revealed different NPY recognition mechanisms in Y_1_R and Y_2_R.

**FIGURE 2:**
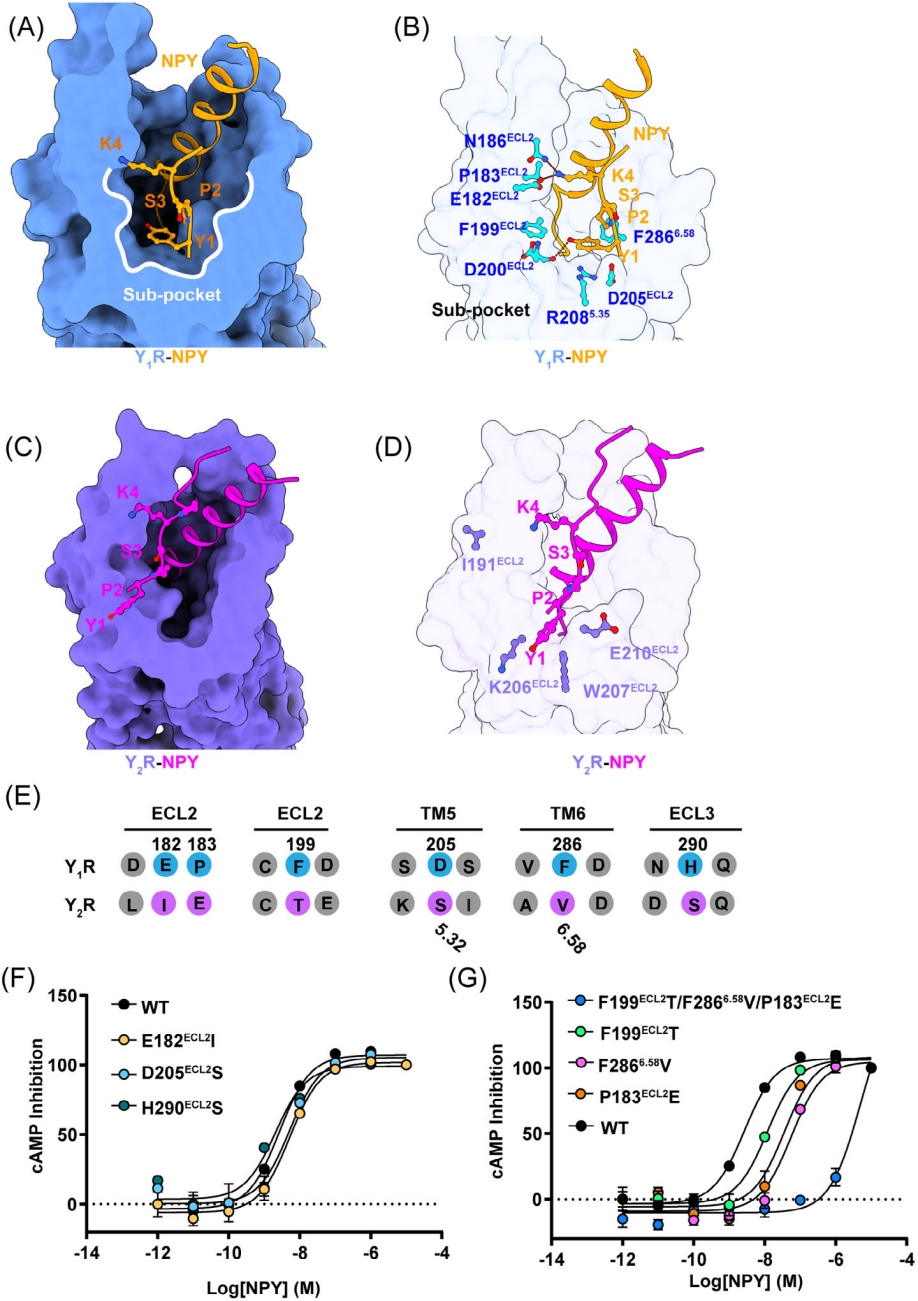
The recognition of N‐terminus of NPY by Y_1_R. (A and B) Cut‐away view of the ligand‐binding pocket of Y_1_R in complex with NPY (top view). The sub‐pocket for the N‐terminus of NPY is shown in detail (B). (C) and (D) Cut‐away view of the ligand‐binding pocket of Y_2_R in complex with NPY (top view). The interactions between NPY and Y_2_R are shown in detail (D). (E) Sequence alignment of the residues forming sub‐pocket inY_1_R and Y_2_R. (F) Concentration–response curves of Y_1_R and polar residues of sub‐pocket mutations in response to stimulation with NPY, respectively. Values are shown as the mean ± s.e.m. of three experiments (*n* = 3) performed in triplicate. (G) Concentration–response curves of Y_1_R and hydrophobic residues of sub‐pocket mutations in response to stimulation with NPY, respectively. Values are shown as the mean ± s.e.m. of three experiments (*n* = 3) performed in triplicate.

In detail, the NPY N‐terminal Y^1^ and P^2^ residues fitted into the amphiphilic cavity formed by hydrophobic (P183^ECL2^, F199^ECL2^, and F286^6.58^) and polar (E182^ECL2^ and D200^ECL2^) residues, and the corresponding [Leu^31^, Pro^34^]–NPY residue adopted a conformation similar to that of NPY (Figure S6A). Therefore, we defined the cavity in Y_1_R as a unique sub‐pocket (Figures 2A and B), where the clusters of hydrophobic residues (P183^ECL2^, F199^ECL2^, and F286^6.58^) formed a hydrophobic contact network with Y^1^ and P^2^ residues and D200^ECL2^ and E182^ECL2^ established a polar network with the NPY peptide Y^1^ and K^4^ side chains (Figure 2B). In addition, two critical residues R208^6.35^ and F286^6.58^ bifurcated the NPY binding pocket in Y_1_R into two cavities, a sub‐pocket, and a major orthosteric pocket (Figure 2B). In contrast to the Y_1_R NPY recognition mode, Y_2_R did not contain the equivalent sub‐pocket for NPY binding (Figures 2C and D).

To further identify the key residues of the Y_1_R sub‐pocket for the NPY N‐terminus recognition, we conducted a series of mutagenesis analyses. The Y_1_R and Y_2_R sequence alignment results confirmed that the Y_1_R sub‐pocket residues (E182^ECL2^, D200^ECL2^, P183^ECL2^, F199^ECL2^, and F286^6.58^) were not conserved (Figure 2E). We replaced these key residues with the corresponding residues in Y_2_R and our mutagenesis test demonstrated that mutations E182I, D205S, and H290S in Y_1_R did not affect the receptor activation potency (Figure 2F and Table S2). However, the hydrophobic residues of sub‐pocket mutations (P183^ECL2^E, F199^ECL2^T, F286^6.58 ^V, and P183^ECL2^E/F199 ^ECL2^T/F286^6.58 ^V) all severely reduced Y_1_R potency to NPY (Figure 2G and Table S2). Our results indicate that the sub‐pocket‐forming hydrophobic residues contribute to the Y_1_R‐mediated NPY recognition.

Compared with other β‐branch structures of class A GPCRs, for instance, the endogenous endothelin ET‐1 peptide‐bound ET_B_ receptor structure revealed a similar peptide–receptor binding mechanism as that in Y_1_R, in which the extracellular part of the receptor (ECL2, TM6, and ECL3) is widely involved in the N‐terminal ET‐1 binding^33^ (Figure S6B). However, the N‐terminal ET‐1 region mainly forms hydrogen bonds and electrostatic interactions with ET‐1 and no such Y_1_R sub‐pocket has been observed in the ET_B_–ET1 structure (Figures S6B–C).

Our structures revealed a sub‐pocket, which plays important roles in the N‐terminal NPY recognition and receptor activation of Y_1_R. Our observation was consistent with previous Y_1_R‐related NPY NMR modeling and docking studies.^37^ Taken together, our study provides insights into the detailed NPY N‐terminus‐Y_1_R interactions and helps in fully understanding the different selectivity mechanisms of various N‐terminally truncated NPY peptides upon Y_1_R and Y_2_R activation.

### 2.3 Adaptive evolution of the Y_1_R sub‐pocket

**FIGURE 3:**
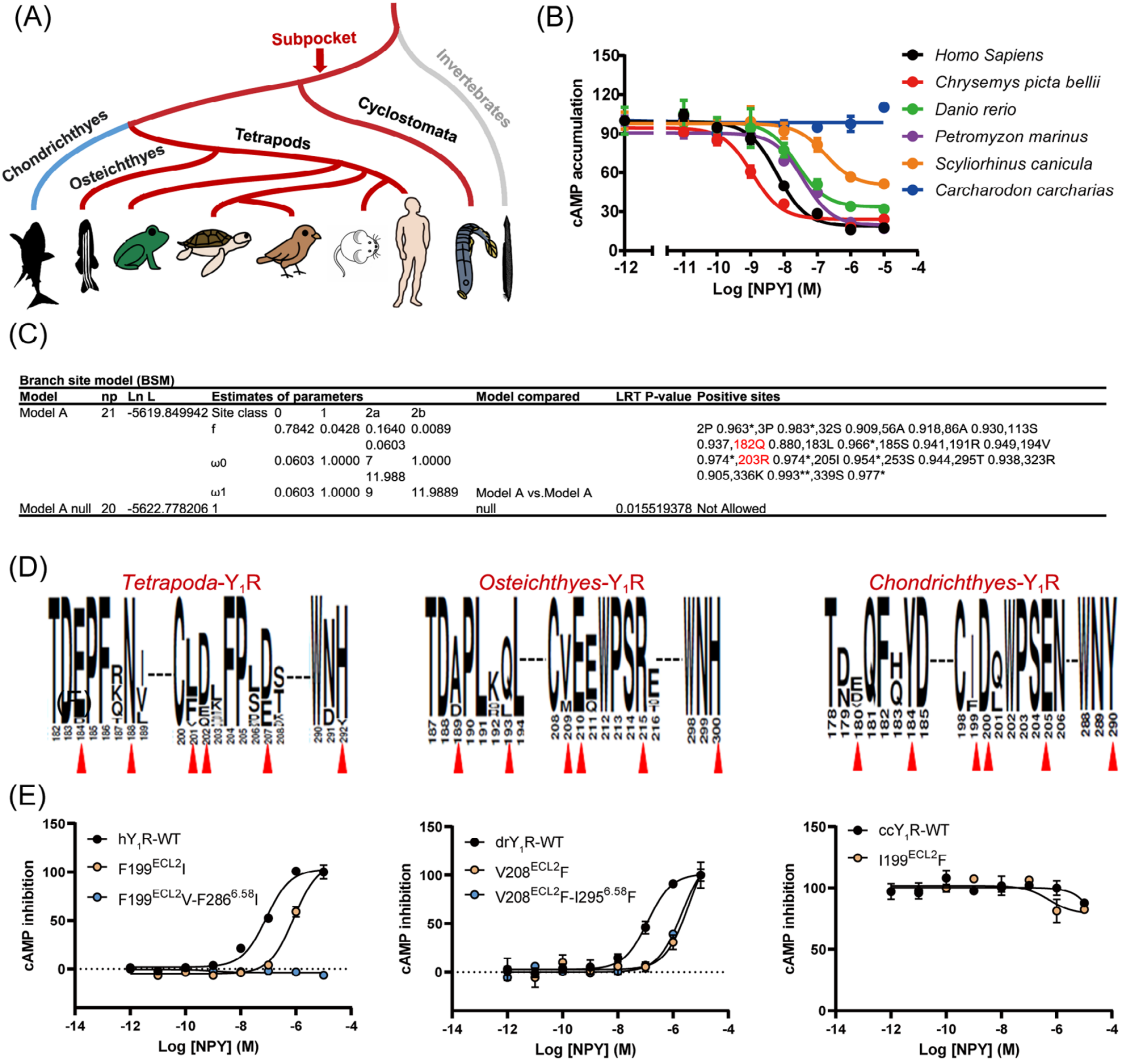
The evolution of Y_1_R in vertebrates. (A) Diagram of Y_1_R in vertebrates and invertebrates. (B) Concentration–response curves of Y_1_R in response to stimulation with NPY in vertebrates. Data represent mean ± s.e.m. from three independent experiments (*n* = 3) performed in triplicate. (C) The parameters and statistical significance of LRTs for branch of Osteichthyes Y_1_R; the dN/dS ratio is calculated with the whole protein coding region; **, *, and no * indicate *p* values in excess of 0.99, 0.95, and 0.90, respectively. (D) Sequence logo of Y_1_Rs in vertebrates. Red triangles represent positions of sub‐pocket sites of Y_1_Rs. (E) Representative of effects of hY1R mutations, drY1R mutations, and ccY1R mutations of the Y_1_R on NPY in cAMP accumulation assays. Data represent mean ± s.e.m. from three independent experiments (*n* = 3) performed in triplicate.

NPY and Y_1_R evolutionary dynamics in early vertebrates have already been thoroughly studied.^2^, ^38^ However, the evolutionary fate of Y_1_R sub‐pocket is elusive. We collected 22 genomes of vertebrates and invertebrates to reveal the related evolutionary processes. Our results revealed that NPY and Y_1_R were present in vertebrates such as Cyclostomata, Chondrichthyes, Osteichthyes, and Tetrapoda, but not in invertebrates (Figure 3A). We selected six classical vertebrate Y1Rs for signal pathway detection and the results demonstrated that Cyclostomata (pmY_1_R/*Petromyzon marinus*), Osteichthyes (drY_1_R/*Danio rerio*), and Tetrapoda (hY_1_R/*Homo Sapiens*, and cpbY_1_R/*Chrysemys picta bellii*) Y1Rs could be activated by NPY, while such activation is significantly weakened (scY_1_R EC_50 _= 175.1 nM and ccY_1_R could not be activated by NPY) in Chondrichthyes (scY_1_R/*Scyliorhinus canicula* and ccY_1_R/*Carcharodon carcharias*) (Figures 3A and B). The branch‐site model has proved to be a useful tool for detecting biological hypotheses of positive selection and generative mutation research and functional analysis.^39^ So, we used a branch‐site model to assess whether the Y_1_Rs underwent positive selection.^40^, ^41^ As shown in Figure 3C, the Y_1_R sequences exhibited a relevant nonsynonymous (dN)/synonymous (dS) substitution rate ratio (branch‐site dN/dS of *ω* > 1; Figures 3A and C) that was highly significant (likelihood ratio tests [LRTs], *p *< 0.05; Figure 3C) only in the Osteichthyes lineage. In contrast, Chondrichthyes did not exhibit this ratio, suggesting that positive Darwinian selection occurred specifically in Osteichthyes, but not in Chondrichthyes Y_1_Rs (Figure 3C). And we found that the sub‐pocket residues Q182 and R203 in Osteichthyes Y_1_R were under positive selection (Figure 3C). Furthermore, according to sequence logos between Osteichthyes and Chondrichthyes Y_1_Rs, the six sub‐pocket residues were non‐conserved (Figure 3D).

To verify the Y_1_R‐related functional differences between Chondrichthyes and other vertebrates, three vertebrate species (human/*Homo Sapiens*, zebrafish/*Danio rerio*, and Carcharodon/*Carcharodon carcharias* representing Tetrapoda, Osteichthyes, and Chondrichthyes, respectively) were selected to test the biological experimental research (Figure 3E). We used the NPY peptide to activate the different Y_1_Rs. NPY activated humans and zebrafish, but not Carcharodon, Y_1_Rs. To further investigate the function of the six residues (E182^ECL2^, Q186 ^ECL2^, F199 ^ECL2^, D200^ECL2^, D205^5.32^, F286^6.58^, and H290^ECL3^) in the sub‐pocket, the human Y_1_R residues (hY_1_R) were mutated to the corresponding residues in the Chondrichthyes orthologs. Similar to the effect of mutating the corresponding key residues in Y_2_R, residue F199^ECL2^ and F286^6.58^ substitutions markedly affected the hY_1_R activation efficacy in response to the NPY peptide (Figure 3E). In addition, residues N186 and D205 in human Y_1_R sub‐pocket under positive selection were also engaged in NPY recognition (Figure S7A). Next, Carcharodon Y_1_R (ccY_1_R) (Q181^ECL2^, Y184 ^ECL2^, I199 ^ECL2^, E205^5.32^, and Y290^ECL3^) and zebrafish Y_1_R (drY_1_R) (A188 ^ECL2^, Q192 ^ECL2^, V208 ^ECL2^, R214^5.32^, and I295^6.58^) residues were reversely mutated to the corresponding hY_1_R. We observed that mutated residues in Carcharodon Y_1_R to the corresponding hY_1_R does not rescue their ability to respond to the NPY. From an evolutionary perspective, the reason is that the protein conformation of Carcharodon Y_1_R has undergone significant changes, and rescuing their ability to respond to NPY may require more than a few point mutations (Figures 3E and S7B‐C). Taken together, our results indicated that the positive selection pressure in Osteichthyes Y_1_R maintains the sub‐pocket and contributes to retaining the Gi signaling. In contrast, Chondrichthyes were not constrained by the selection pressure, and the mutations in the sub‐pocket amino acids led to changes in their Gi signaling.

### 2.4 Conserved common site for NPY recognition by NPYRs

Overall, the NPY‐bound Y_1_R and Y_2_R structures shared a similar conformation and central residues, E15–L31 (α‐helix region) of NPY formed approximately five α‐helical turns and the base of the NPY α‐helix overlay in the two structures, while the amino terminus of the Y_1_R helix was rotated approximately 10˚ inwards compared with that in Y_2_R (Figure S8A). The residue R^25^ of NPY in the Y_1_R structure formed hydrogen bonds with D104^2.68^, which could not be observed in the NPY‐Y_2_R structure, potentially contributing to the inwards shift of the NPY‐bound Y_1_R α‐helix (Figure S8B). In addition, the hydrogen bonds between the residue R^25^ of NPY and D104^2.68^ further stabilized the NPY–Y_1_R interactions. Consistent with these structural observations, a D104A^2.68^ substitution reduced the potency of the Y_1_R response for NPY (∼73 folds) (Figure S8C).

**FIGURE 4:**
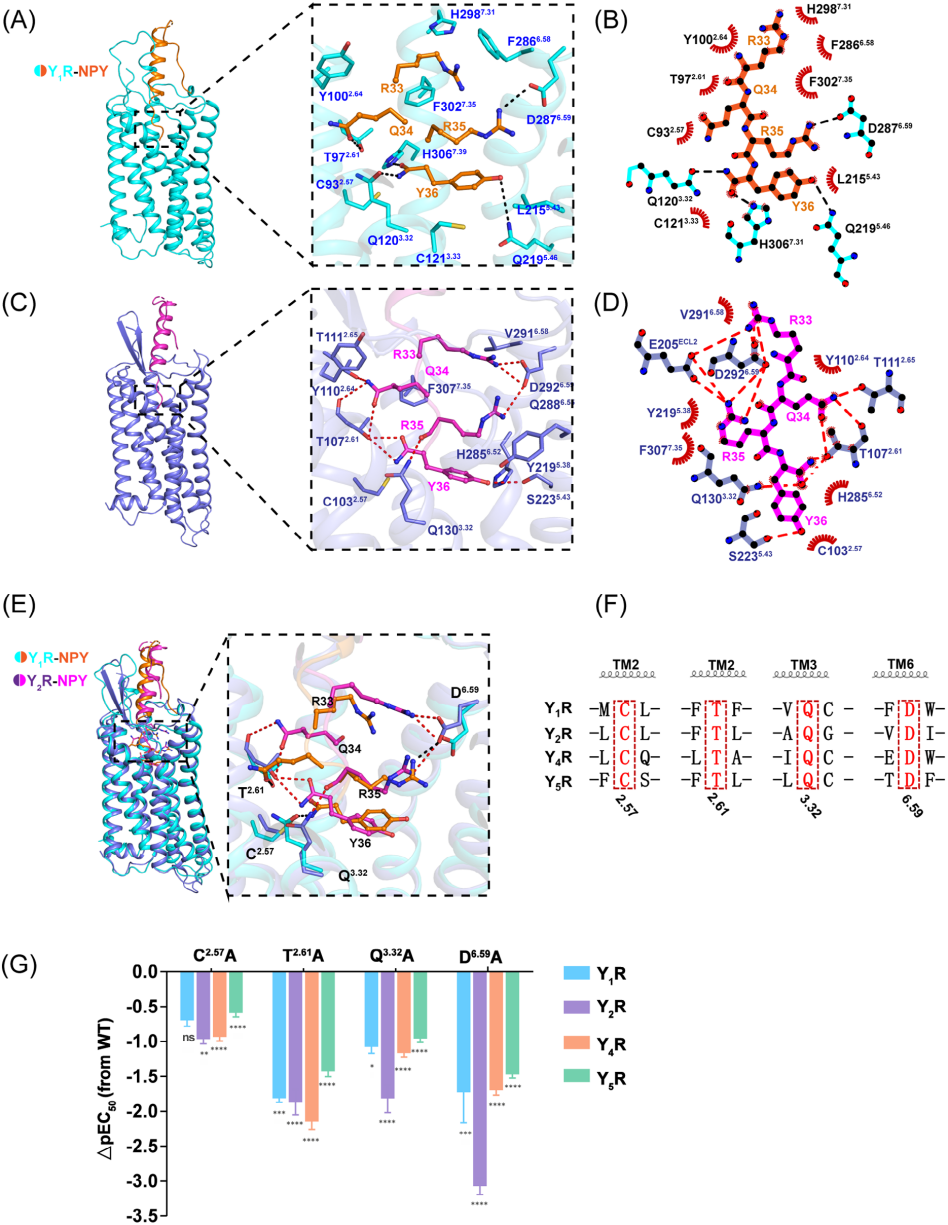
Conserved common sites for C‐terminus of NPY recognition by NPYRs. (A) Ligand‐binding pocket for the C‐terminus of NPY. Y_1_R is shown in cyan cartoon representation. The Y_1_R residues that form interactions with the C‐terminus of NPY are shown as sticks. The C‐terminus residues (R^33^‐Q^34^‐R^35^‐Y^36^‐NH_2_) of NPY (carbon in orange) is shown as sticks and hydrogen bonds are shown as black dashed lines. (B) Schematic representation of interactions between Y_1_R and the C‐terminus residues (R^33^‐Q^34^‐R^35^‐Y^36^‐NH_2_) of NPY analyzed using LigPlot+ program.^50^ The Y_1_R residues engaged in hydrogen bonds are shown as cyan sticks. Hydrogen bonds are shown as black dashed lines. (C) Ligand‐binding pocket for the C‐terminus of NPY. Y_2_R is shown in medium slate cartoon representation. The Y_2_R residues that form interactions with the C‐terminus of NPY are shown as sticks. The C‐terminus residues (R^33^‐Q^34^‐R^35^‐Y^36^‐NH_2_) of NPY (carbon in magenta) is shown as sticks and hydrogen bonds are shown as red dashed lines. (D) Schematic representation of interactions between Y_2_R and the C‐terminus residues (R^33^‐Q^34^‐R^35^‐Y^36^‐NH_2_) of NPY analyzed using LigPlot+ program. The Y_2_R residues engaged in hydrogen bonds are shown as medium slate sticks. Hydrogen bonds are shown as red dashed lines. (E) Comparison of C‐terminus of NPY binding modes between Y_2_R and Y_1_R. (F) Sequence alignment of the conserved residues for C‐terminus of NPY recognition in Y_1_R and Y_2_R. (G) NPY‐induced cAMP accumulation assays of the conserved common sites in Y_1_R, Y_2_R, Y_4_R, and Y_5_R. Bars represent differences in calculated NPY potency [pEC_50_] for each mutant relative to the wild‐type receptor (WT). Values are shown as the mean ± s.e.m. of three experiments (*n* = 3) performed in triplicate. *P* Values were determined by one‐way of variance ANOVA with Dunnett's test. *p < 0.05; **p < 0.01; ***p < 0.001; ****p ≤ 0.0001; ns, no significant difference.

NPY in the Y_1_R and Y_2_R complex structures adopted similar conformations in the recognition by the NPYR orthosteric pockets (Figures 4A–D). The C‐terminus of NPY (residues 33−36), which was confirmed to display major importance for all NPYR bindings,^3^, ^42^ adopted an extended conformation and reaches far into the cores of Y_1_R and Y_2_R, contacting all TM helices except for TM1, as well as residues in ECL2 and ECL3 through an extensive interface of hydrophobic and polar interactions (Figures 4A–D). The NPY C‐terminal‐bound Y_1_R structure exhibited both important common characteristics and notably distinct features compared to the NPY‐bound Y_2_R structure (Figures 4A–D). The structural comparison revealed that the Y_1_R‐bound NPY side chains R^35^ and Y^36^ overlaid well with those of Y_2_R‐bound NPY, while the NPY residues R^33^ and Q^34^ occupied different binding sites to those of Y_2_R‐bound NPY (Figure 4E).

At the bottom region of the orthosteric peptide‐binding pocket, polar receptor residues formed an extensive polar interaction network with amidated Y^36^ both in the Y_1_R‐and Y_2_R‐bound NPY structures, structurally supporting the fact that this amidation modification was necessary for the NPYR activity.^43^, ^44^ The Y^36^ amidation group and hydrogen bond established polar contacts with residues T107^2.61^ and S223^5.46^ in the NPY‐Y_2_R structure (Figures 4C and D), whereas the amidation and carbonyl groups of Y^36^in the Y_1_R‐bound NPY formed hydrogen bonds with residues Q120^3.32^ and H306^7.39^, respectively (Figures 4A and B). In addition, residue Y^36^ was further coordinated by Q219^5.46^ through polar interactions and C93^2.57^ through van der Waals contacts in the NPY‐Y_1_R structure (Figures 4A and B). On the same Y^36^ orientation, residue R^35^ was fastened mainly through polar interactions by D^6.59^ in the NPY–Y_1_R and NPY–Y_2_R structures (Figures 4A–D). Notably, previous studies reported that the ionic interaction of residue D^6.59^ is key for positively charged ligand recognition.^45^

To further identify the common binding sites NPYRs, we conducted a series of mutagenesis analyses. Our NPYR sequence alignments indicated that residues (C^2.57^, T^2.61^, Q^3.32^, and D^6.59^) in TM2, TM3, and TM6, which form polar interactions and van der Waals contacts with the C‐terminus residues Y^36^ and R^35^, were conserved among the four receptor subtypes (Figures 4E and F). Mutation of these conserved residues (C^2.57^A, T^2.61^A, Q^3.32^A, and D^6.59^A) in Y_1_R, Y_2_R, Y_4_R, and Y_5_R significantly impaired receptor activity (Figures 4G, S9–12 and Table S2), supporting the essential role of these residues in NPY recognition. Taken together, the structural observations and mutagenesis analyses revealed that NPYRs share common binding sites to bind NPY residues Y^36^ and R^35^ and adopt different molecular patterns in the interaction with NPY.

### 2.5 The different recognition patterns in Y_1_R and Y_2_R

**FIGURE 5:**
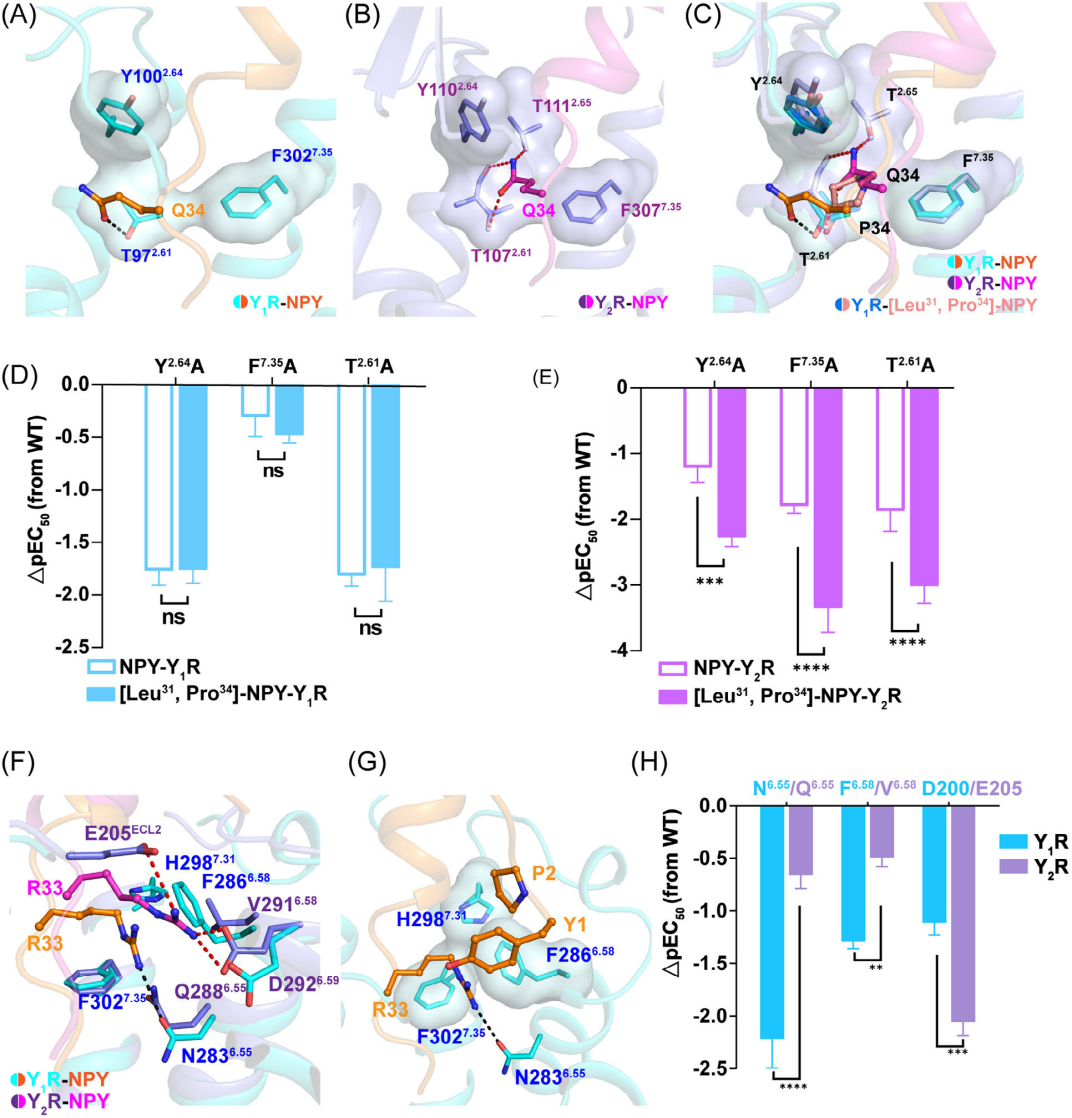
The selectivity for NPY and [Leu^31^, Pro^34^]–NPY recognitions by Y_1_R and Y_2_R. (A) Detailed interactions between C‐terminus residues Q^34^ (sticks, orange) of NPY and Y_1_R (cyan) are shown. The residues interacting with Q^34^ of Y_2_R are shown as sticks and surface. The polar contacts are shown as black dashed lines. (B) Detailed interactions between C‐terminus residues Q^34^ (sticks, magenta) of NPY and Y_2_R (medium slate) are shown. The residues interacting with Q^34^ of Y_2_R are shown as sticks and surface. The polar contacts are shown as red dashed lines. (C) Comparison of the detailed interactions between C‐terminus residues Q^34^ (sticks, orange, magenta) of NPY or P^34^of [Leu^31^, Pro^34^]–NPY (sticks, light salmon) and residues of Y_1_R and Y_2_R. (D and E) NPY‐induced or [Leu^31^, Pro^34^]–NPY‐induced cAMP accumulation assays of the sites in Y_1_R (D) and Y_2_R (E). Bars represent differences in calculated NPY or [Leu^31^, Pro^34^]–NPY potency [pEC_50_] for each mutant relative to the wild‐type receptor (WT). Values are shown as the mean ± s.e.m. of three experiments (*n* = 3) performed in triplicate. *p* Values were determined by unpaired *t*‐test. **p < 0.01; ***p < 0.001; ****p ≤ 0.0001; ns, no significant difference. (F) Comparison of the detailed interactions between C‐terminus residues R^33^ (sticks, orange, magenta) of NPY and residues (sticks, cyan, medium slate) of Y_1_R and Y_2_R. The residues interacting with Q^33^ of Y_1_R and Y_2_R are shown as sticks and surface. (G) The detailed interactions between C‐terminus residues R^33^ (sticks, orange) of NPY and residues (sticks, cyan) of Y_1_R. (H) NPY‐induced cAMP accumulation assays of the sites in Y_1_R and Y_2_R. Bars represent differences in calculated NPY potency [pEC_50_] for each mutant relative to the wild‐type receptor (WT). Values are shown as the mean ± s.e.m. of three experiments (*n* = 3) performed in triplicate. *p* Values were determined by unpaired *t*‐test. **p < 0.01; ***p < 0.001; ****p ≤ 0.0001.

Our cAMP assay data demonstrated [Leu^31^, Pro^34^]–NPY retained similar activation potency as NPY on Y_1_R, but displayed higher selectivity for Y_1_R over Y_2_R, indicating that both Y_1_R and Y_2_R has different mechanisms for ligand recognition. Our structures of Y_1_R and Y_2_R in complex with NPY or [Leu^31^, Pro^34^]–NPY offer templates to understand the ligand recognition or selectivity. In the case of the NPY‐Y_1_R structure, the residue Q^34^ is observed to interact with only T97^2.61^ in Y_1_R through hydrogen bonding (Figure 5A). Whereas the side chain of Q^34^ in Y_2_R displayed a different conformation and was projected into a cavity shaped by TM2 and TM7, interacting with T107^2.61^, Y110^2.64^, T111^2.65^, and F307^7.35^ in Y_2_R (Figures 5B and S13A).

To further analyze the selectivity of [Leu^31^, Pro^34^]–NPY, we determined the structure of the [Leu^31^, Pro^34^]–NPY‐bound Y_1_R in complex with the Gi2 protein. The C‐terminal parts of NPY and [Leu^31^, Pro^34^]–NPY adopted similar binding pose in the orthosteric pocket of Y_1_R (Figure S13B). Residue P^34^ of [Leu^31^, Pro^34^]–NPY share a similar position with residue Q^34^ of NPY (Figures 5C and S13B and C). The substitution of Q with P at position 34 of NPY disrupted the interactions between Q34 and T97^2.61^ in Y_1_R. The residue P^34^ in [Leu^31^, Pro^34^]–NPY peptide was not observed to make polar interaction with Y_1_R, and this kind of peptide did not influence Y_1_R signaling activation. Comparing to structures of Y_1_R bound NPY or [Leu^31^, Pro^34^]–NPY, the residue Q^34^ of NPY makes hydrogen bonds with T107^2.61^ and T111^2.65^ in Y_2_R (Figure 5C). These residues T107^2.61^, Y110^2.64^, T111^2.65^, and F307^7.35^ that involved in ligand binding pocket are conserved in both Y_1_R and Y_2_R. We next investigated whether these conserved residues play different roles in Y_1_R and Y_2_R, the residues T^2.61^, Y^2.64^, T^2.65^, and F^7.35^ were replaced with Ala both in Y_1_R or Y_2_R. The results of our functional assays showed that all mutants of Y_2_R significant lost NPY‐induced signal transduction, which further confirm the important roles of these residues in Y_2_R (Figure S13D). By contrast, T^2.65^A and F^7.35^A mutations in Y_1_R displayed similar activation potency as wild‐type receptor in response to NPY (Figure S13D).

To further compare the pharmacological featured of NPY and [Leu^31^, Pro^34^]–NPY, we measured the NPY or [Leu^31^, Pro^34^]–NPY‐induced Y_1_R and Y_2_R activation. For Y_1_R, NPY and [Leu^31^, Pro^34^]–NPY displayed similar potencies on Y_1_R or mutants (Figure 5D). However, both NPY analogs exhibited different activation potencies for Y_2_R and its mutants (Figure 5E).

In addition, structural comparisons of Y_1_R and Y_2_R revealed a notable displacement of R^33^ from NPY peptides in two receptors (Figure 5F). In Y_2_R structure, the residue R^33^ is observed to interact with D292^6.59^ and forms polar interactions with R^35^ and E205^ECL2^ via hydrogen bonding (Figure 5F). In Y_1_R structure, the side chain of R^33^ in NPY is found to insert a cavity shaped by TM6 and TM7, establishing hydrophobic contacts with residues F302^7.35^, H298^7.31^, and F286^6.58^ in Y_1_R (Figure 5F). Consistent with the structural observations, the results of mutagenesis studies and functional assays showed that the mutation E205^ECL2^A influenced NPY induced Y_2_R activation potency, whereas the mutation N283^6.55^A reduced the Y_1_R activation induced by NPY (Figures 5H, S9–12 and Table S2). Notably, the residues F286^6.58^ in Y_1_R appears to be a key facet to shape the sub‐pocket (Figure 5G).

### 2.6 Structural basis of NPY mediated Y_1_R and Y_2_R activation

**FIGURE 6:**
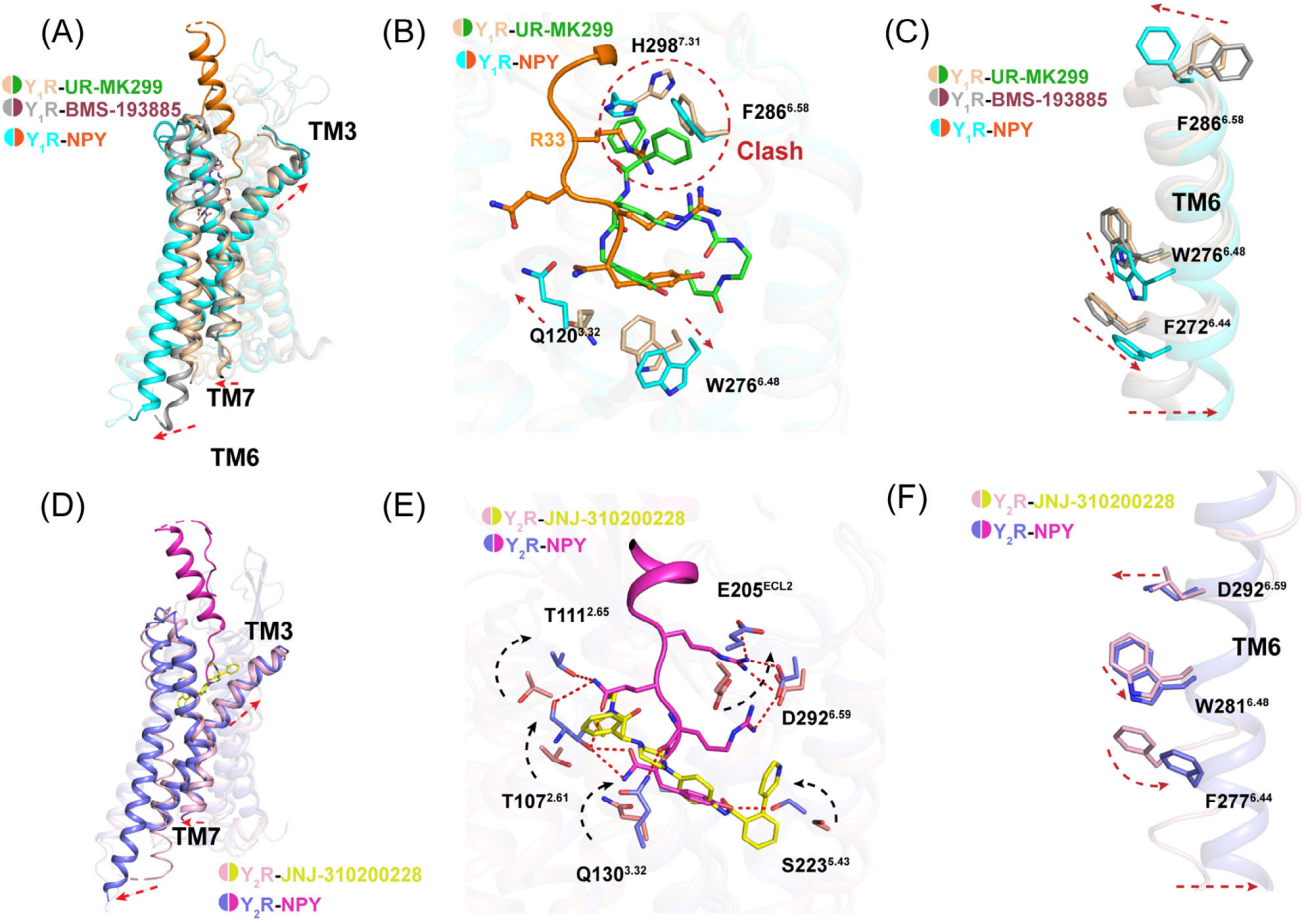
Mechanism of Y_1_R and Y_2_R activation by NPY binding. (A) Structural superposition of the active and antagonist‐bound Y_1_R. (B) Conformational changes comprising of F286^6.58^, H298^7.31^, and F302^7.35^ upon peptide activation. UR‐MK299 push the side‐chain of F286^6.58^ to swing away from the receptor helical core due to steric clash. The swing orientations of F286^6.58^, H298^7.31^, and F302^7.35^ were indicated by red arrows. The clashes were highlighted as red oval dashed lines. (C) Conformational changes of Toggle switch of the conserved “micro‐switches” upon Y_1_R activation. The conformational changes of residue side chains and the outward displacement of TM6 are shown as red arrows upon receptor activation. (D) Structural superposition of the active and antagonist‐bound Y_2_R. (E) Conformational changes comprising of residues interacting with NPY upon peptide activation. D^6.59^ displays upon movement, shown as a black arrow. (F) Conformational changes of the toggle switch in conserved “micro‐switches” upon receptor activation. The conformational alterations of residue side chains are depicted with red arrows following receptor activation. Additionally, the outward displacement of TM6 in the active receptor is illustrated by a red arrow.

The structural comparison of our two Gi2‐coupled NPY receptors with other class A GPCRs revealed similar conformations.^46^ TM6 and TM7 of Y_1_R and Y_2_R adopted nearly identical conformations to the active structures of the neurotensin receptor NTS1,^47^ orexin receptors,^35^ and the endothelin ET_B_ receptor^33^ (Figure S14). Furthermore, the structural comparison of two Y_1_R and Y_2_R receptors with the antagonist‐bound NPYRs (PDB ID: 5ZBH, 5ZBQ, and 7DDZ),^31^, ^32^ supports the contention that these two complexes were in the active state (Figures 6A and D). Compared with structure of antagonist BMS‐193885 bound Y_1_R (PDB ID: 5ZBH), the activated Y_1_R complex displayed pronounced outward displacements (∼8 Å, measured at Cα of T258^6.30^) of the TM6 at cytoplasmic region, and ∼5 Å inward shift of TM7 (measured at Cα of Y320^7.53^) (Figure 6A).

The residue F286^6.58^ in Y_1_R was demonstrated to play important role in receptor activation as well as ligand selectivity. The detailed comparison of both active and inactive structures reveals a significant movement of F286^6.58^ toward receptor helical core, which may initiate the cascade of conformational changes upon agonist binding. Meanwhile, the microswitch residue W^6.48^ is observed to display rotameric change (Figures 6C and F), synergistically, F^6.44^ in P‐I‐F motif underwent conformational changes to facilitate G‐protein coupling (Figure S15). In addition, residue Q^3.32^ conserved in Y_1_R and Y_2_R formed hydrogen bonds with NPY, leading to a slight upward movement of TM3 (Figures 6A, B, D, and E).

## 3 DISCUSSION

NPY serves a critical role in modulating a variety of physiological processes in both the central nervous system and peripheral tissues. It exerts its effects through binding to G protein‐coupled NPY receptors, with the Y_1_ and Y_2_ receptor subtypes being particularly noteworthy. Gaining a comprehensive understanding of the structural mechanisms through which Y_1_R and Y_2_R interact with NPY is indispensable for the rational design of selective drugs.

Although it reported some structures of the NPY‐bound receptor complex^48^, ^49^ during the preparation of our manuscript, some new insights about ligand selectivity mechanisms of NPYRs were still obtained in our research. Here, we present three cryo‐EM structures of Gi2‐coupled Y_1_R and Y_2_R bound to either NPY or [Leu^31^, Pro^34^]–NPY. These structures reveal a conserved orthosteric peptide‐binding pocket in both Y_1_R and Y_2_R that interacts with the C‐terminal region of NPY. The extreme C‐terminal dipeptide with amidated modification (R^35^‐Y^36^‐NH_2_) is buried in the bottom of orthosteric pocket, which is highly conserved between the two NPYR subtypes. Through a combination of structural observations and alanine mutagenesis analysis, we demonstrate that the binding of the NPY C‐terminus is critical for the activation potency or efficacy of NPY.

In contrast, distinct physiochemical environments surrounding a tetrapeptide (Y^1^‐P^2^‐R^33^‐Q^34^) between Y_1_R and Y_2_R serve as determinants for two NPYR subtype preference. Intriguingly, we identified a distinct sub‐pocket in Y_1_R that contributes to ligand selectivity and the sub‐pocket of Y_1_R plays an essential role in the recognition of the N‐terminus of NPY and receptor activation. Notably, Y^1^‐P^2^‐R^33^ forms a specific interaction network with hydrophobic residues F302^7.35^, H298^7.31^, F286^6.58^, enhancing the Y_1_R selectivity.

Moreover, comparisons of the NPY‐ Y_1_R, NPY–Y_2_R, and [Leu^31^, Pro^34^]–NPY–Y_1_R complex structures highlight the plasticity of orthosteric pockets across receptor subtypes. Substituting Q^34^ with P^34^ disrupts the original polar and hydrophobic interactions with Y_2_R, which plays a more indispensable role of Y_2_R to interact with NPY than Y_1_R, thus leading to the Y_2_R selectivity.

In summary, we offer detailed molecular maps depicting the binding of NPY peptides to various NPY receptor subtypes, thereby shedding light on subtype‐specific interaction patterns. This critical insight substantially broadens our knowledge of ligand recognition and signal transduction within the NPY–GPCR system. Consequently, our findings establish a robust foundation for the future development of selective drugs aimed at specific NPY receptor targets, thereby paving the way for more precise and efficacious therapeutic strategies.

## 4 MATERIALS AND METHODS

The details of the protein expression and purification, cryo‐EM structure determination, and pharmacological experiments are provided in Supplementary Information Methods.

## AUTHOR CONTRIBUTIONS

W. Y. and Z. S. initiated structural studies of NPYRs and their ligands. S. S. and C. S. designed the expression constructs, purification, and preparation of the NPY–Y_1_R–Gi2, NPY–Y_2_R–Gi2 and [Leu^31^, Pro^34^] NPY–Y_1_R–Gi2 complexes. S. S., L. C., and C. Z. carried out cryo‐EM screening, data collection, and model building and refinement in the study. Y. D., C. W., and K. W. performed functional assays. Z. Y. and H. H. contributed in purification of scFv16. H. C. performs bioinformatics analysis under the direction of C. D. S. S. and Y. D. prepared figures. S. S., C. S., and Y. D. planned and coordinated the entire project under the supervision of W. Y. and Z. S. W. Y., F. Y., C. D., and Z. S. supervised the overall project and wrote the manuscript. All authors have read and approved the final manuscript.

## CONFLICT OF INTEREST STATEMENT

The authors declare no competing interest.

## ETHICS STATEMENT

Not applicable.

## Supporting information

Supporting Information

## Data Availability

Structural data from this study have been deposited in the Protein Data Bank (PDB) under coordinate accession numbers 8K6M (cryo‐EM structure of the NPY–Y_1_R–Gi2 complex), 8K6O (cryo‐EM structure of the [Leu^31^, Pro^34^]–Y_1_R–Gi2 complex) and 8K6N (cryo‐EM structure of the NPY–Y_2_R–Gi2 complex). Additionally, the Electron Microscopy Data Bank (EMDB) accession numbers EMD‐36923, EMD‐36925, and EMD‐36924 correspond to the cryo‐EM structures mentioned above. All remaining data generated or analyzed during this research are available within the published article and its Supplementary Information files.
