# Supplementary material for: Structural basis of neuropeptide Y signaling through Y1 and Y2 receptors
Source: MedComm (2020). 2024 Jun 15;5(7):e565. doi: 10.1002/mco2.565 (PMC11179954; doi:10.1002/mco2.565)
Supplement: Supplementary file 1 — Supporting Information [file MCO2-5-e565-s001.pdf]

Supplementary information

Structural basis of neuropeptide Y signaling through Y_1 and Y_2 receptors

Siyuan Shen[1,2,6], Yue Deng[1,6], Chenglong Shen[1,6], Haidi Chen[3,6], Lin Cheng[4,6], Chao Wu[1,6], Chang Zhao[1,6], Zhiqian Yang[1], Hanlin Hou[1], Kexin Wang[1], Zhenhua Shao[1,2*], Cheng Deng[3*], Feng Ye[5*] and Wei Yan[1*]

[1]Division of Nephrology and Kidney Research Institute, State Key Laboratory of Biotherapy, West China Hospital, Sichuan University, 610041 Chengdu, Sichuan, China.

[2]Frontiers Medical Center, Tianfu Jincheng Laboratory, Chengdu, 610212, China. Institutes for Systems Genetics, Frontiers Science Centre for Disease-Related.

[3]Institutes for Systems Genetics, Frontiers Science Centre for Disease-Related Molecular Network, West China Hospital, Sichuan University, Chengdu 610212, Sichuan, China.

[4]Department of Otolaryngology Head and Neck Surgery, Sichuan Provincial People's Hospital, University of Electronic Science and Technology of China, Chengdu 610000, China.

[5]Department of Pathology, Institute of Clinical Pathology, Frontiers Science Center for Disease-related Molecular Network, West China Hospital of Sichuan University, Chengdu, China.

[6]These authors contributed equally.

*Correspondence: zhenhuashao@scu.edu.cn (Z.S.); dengcheng@wchscu.cn(C.D.), fengye@scu.edu.cn (F.Y.), weiyan2018@scu.edu.cn (W.Y.).

Supplementary Methods

Constructs

The full-length of wild-type human Y_1R and Y_2R gene were cloned into the pFastBac1 vector with the haemagglutinin (HA) signal sequence followed by a Flag tag and T4 lysozyme (T4L) tags at the N-terminal. The wild-type human Gαi2 (UniProt: P04899) was inserted into the pFastBac1 vector. The human Gβ1 with an N-terminal 6×hexahistidine (His)-tag and human Gγ2 were inserted into a pFastBac-dual vector.

Expression and purification of scFv16 antibody

scFv16 was expressed and purified according to a previously described method[1]. Briefly, scFv16 was subcloned into pfastBac1 with the C-terminal 6×hexahistidine (His)-tag and the N-terminal GP67 signal peptide. 6×His-tagged scFv16 was expressed using the *Spodoptera frugiperda* Sf9 baculovirus system and purified through Ni-NTA resin. The eluted protein was subsequently concentrated to 2mg/ml in a buffer containing100 mM NaCl and 20 mM HEPES, pH 7.5. Then the protein rapidly frozen in liquid nitrogen and was stored at −80°C for subsequent use.

Expression and purification of Y_1R and Y_2R-Gi complex

The Y_1R and Y_2R-Gi complex was expressed by co infecting the HA-Flag-Y_1R/Y_2R, $G\alpha i_2$, and $G\beta_1G\gamma_2$ baculovirus at a ratio of 1:2:1 into Sf9 cells at a density of 2.5×10^6 cells/ml and cultivated for an additional 48 hours to collect the cell pellets. Cell pellets were lysed with 50 mM NaCl, 20 mM HEPES pH 7.5, 10 mM $MgCl_2$, Protease Inhibitor Cocktail (100 μg/mL leupeptin, 160 μg/mL benzamidine), 10 μM NPY peptides, 40 μg/mL scFv16, and 50 mU/mL apyrase (NEB) at room temperature for 2h and then centrifuged at 20,000 × g for 30 minutes at 4 °C. The membranes fractions were solubilized with 1% (w/v) lauryl maltose neopentyl glycol (LMNG; Anatrace) supplemented with 50 mM NaCl, 20 mM HEPES pH 7.5, 0.1% (w/v) cholesteryl hemisuccinate (CHS), 10 mM $MgCl_2$, Protease Inhibitor Cocktail, 50 mU/mL apyrase (NEB) and 10 μM NPY peptides for 2 hours at 4 °C. After centrifuging at 65,000×g for

30min, the supernatant was incubated with M1 anti-Flag antibody coupled Sepharose Resin for 2 hours at 4 °C. The target complex was eluted in 100 mM NaCl, 20 mM HEPES pH 7.5, 0.01% LMNG, 0.0033% glyco-diosgenin (GDN; Anatrace), 0.001% CHS, 5 mM EDTA, Protease Inhibitor Cocktail, 100 μM TCEP, 0.2 mg/mL FLAG peptide and 10 μM NPY peptide. The concentrated sample was loaded to a Superose 6 Increase 10/300 GL size exclusion column (GE Healthcare) that was pre-equilibrated in 100 mM NaCl, 20 mM HEPES, pH 7.5, 3 mM $MgCl_2$, 0.00075% (w/v) LMNG, 0.0001% (w/v) CHS, 0.00025% GDN, 5 μM NPY peptide and 100 μM TCEP. Peak fractions were concentrated using an Amicon Ultra Centrifugal Filter (MWCO, 100 kDa).

Cryo-EM grid preparation and data collection

3μL of NPY-Y_1R-Gi2-scFv16, NPY-Y_2R-Gi2-scFv16 and [Leu^{31}, Pro^{34}]-NPY-Y_1R-Gi2-scFv16 complexes at ~10 mg/ ml was applied onto glow-discharged 300-mesh Au grids (Quantifoil R1.2/1.3), respectively. The grids were blotted for 2-3 s and then rapidly frozen in liquid ethane using a Vitrobot Mark IV (Thermo Fisher) at 4 °C and 100% humidity.

For the dataset of NPY-Y_2R-Gi2-scFv16 and [Leu^{31}, Pro^{34}]-Y_1R-Gi2-scFv16 complexes, cryo-EM data were collected on a Titan Krios electron microscope (Thermo Fisher Scientific) equipped with a K2 Summit detector (Gatan) at an accelerating voltage of 300 kV. The data collections were operated at a nominal magnification of 165,000 × in nanoprobe TEM mode. Movies contains 30 frames were recorded for 6s in counting mode by EPU software (Thermo Fisher Scientific), corresponding to a pixel size of 0.85 Å. The total dose of NPY-Y_2R-Gi2-scFv16 and [Leu^{31}, Pro^{34}]-Y_1R-Gi2-scFv16 are 61 and 64 $e^-/Å^2$, respectively.

For the dataset of NPY-Y_1R-Gi2-scFv16 complex, cryo-EM data were collected on a Titan Krios electron microscope (Thermo Fisher Scientific) at 300 kV. The automatic data collection was performed at a nominal magnification of 130,000 × (corresponding to a raw pixel size of 0.46 Å for the micrographs) by a K3 Summit detector (Gatan) with an energy filter (operated with a slit width of 40 eV; GIF) by

using SerialEM software [2]. The movies were recorded with the dose rate of 20 e^-/pix/s and the exposure time is 2.78 s, yielding 36 frames per stack and an accumulated dose of 66 e^-/Å^2.

Image processing and 3D reconstruction

For the dataset of NPY-Y_1R-Gi2-scFv16 complex, a total of 6472 movie stacks were collected and subjected to beam-induced motion correction by MotionCor2[3] and CTF estimation by GCTF [4]. Micrographs with resolution lower than 4 Å were removed and the remaining 4739 micrographs are selected for auto-picking, an initial set 6,072,643 particles were picked and subjected to 2D classification for 3 rounds in RELION3.0 [5], the well-defined 2D-average were selected and extracted with the box size of 256 pixels, subsequently processed for ab-initio reconstruction followed by heterogeneous refinement in cryoSPARC[6]. A good subset containing 737,665 particles were re-imported into RELION3.0[5] followed by a round of 3D classification focusing on the complex excluding the disorder micelle region. A subset of 127,586 particles were subjected to Bayesian polishing and 3D auto-refinement, producing a map with global resolution of 3.5 Å according to the 0.143 criterion of the FSC.

For the dataset of NPY- Y_2R-Gi2-scFv16 and [Leu^{31}, Pro^{34}]-Y_1R-Gi2-scFv16, a total of 5023 and 5112 movie stacks were collected and processed, respectively. The reconstruction procedure was similar to that of NPY-Y_1R-Gi2-scFv16 complex, several rounds of particle picking and classification, the well-defined particles were subjected to 3D auto-refinement yielding a final global map at 3.2 and 3.3 Å, respectively. The final maps were sharpened by DeepEMhancer [7]. The local resolution was estimated in RELION3.0 [5].

Model building and structure refinement

The initial structure of Y_1R (PDB: 5ZBQ)[8] and Y_2R (PDB: 7DDZ)[9] in inactive state was used to build the initial model of Y_1R and Y_2R receptor, respectively. The initial Gi_2 and scFv16 models were created using the cryo-EM structure of S1PR3-Gi1 complex (PDB code: 7EW3) [10]. UCSF Chimera1.14 was used to fit all models into the

EM density map[11], the coordinates were subsequently manual adjustment and rebuilding in COOT [12]. Real space refinement was performed in Phenix program [13]. The model statistics were finally validated by using MolProbity [14] in Phenix. The refinement statistics are presented in supplemental information table S1. UCSF Chimera[11], Chimera X[15] and PyMOL (https://pymol.org/2/) were used to generate the structural figures.

Cyclic adenosine 3′,5′-monophosphate (cAMP) assay.

To measure the ligand-induced Gi signaling activation potency of wild-type NPYR or mutants, the GloSensor cAMP assay (Promega) was conducted following a previous study. Briefly, HEK293 cells were co-transfected with the GloSensor plasmid and various NPYR constructs or pcDNA3.1 (as a negative control) plasmids using Polyethylenimine Linear (PEI, YEASEN) in 6-well plates. Following a 4-hour incubation period, the cells were transferred to fresh medium. After 24 hours, they were harvested and suspended in assay buffer (HBSS with 10 mM HEPES, pH 7.4), supplemented with a 3% v/v dilution of D-Luciferin-Potassium Salt (YEASEN). Following a subsequent 1-hour incubation, ligands, diluted in assay buffer with forskolin, were introduced to the cells. Subsequently, the cells were incubated for 30 minutes in a light-free environment at room temperature Fluorescence signals were measured using a Synergy H1 microplate reader (BioTek). GraphPad Prism 8 (GraphPad Software) was used to processing data using the nonlinear regression dose-response function. Results were represented as the mean ± s.e.m. from three independent experiments. All experiments were repeated at least three times.

Enzyme-linked immunosorbent assay (ELISA)

To evaluate the expression level of wild-type or mutations NPYRs (NPY_1R and NPY_2R), the HEK293 cells in six-well plates were transiently transfected with the corresponding plasmids using Polyethylenimine Linear (PEI, YEASEN). After grown in a 37°C incubator with 5% CO_2 for 24 h, the cells were havested and distributed into

poly-d-lysine-coated 96-well microplates.The next day, 4% (w/v) paraformaldehyde was added to fixed the transfected cells. Subsequently, the cells were blocked with 5% (w/v) BSA at room temperature for 60 min, followed by incubation of anti-Flag HRP conjugated monoclonal antibody (1:2,000, Sigma-Aldrich) overnight at 4°C. After three washes with PBS, the HRP substrate 3,3′,5,5′-tetramethylbenzidine (TMB) were added into each well. The ELISA assay was quenched by adding an equal volume of 1 M HCl solution and the luminescence was counted at 450 nm using the Synergy H1 microplate reader (BioTek).

References

1 Xu, Z. *et al.* Ligand recognition and G-protein coupling of trace amine receptor TAAR1. *Nature* **624**, 672-681, doi:10.1038/s41586-023-06804-z (2023).

2 Mastronarde, D. N. Automated electron microscope tomography using robust prediction of specimen movements. *J Struct Biol* **152**, 36-51, doi:10.1016/j.jsb.2005.07.007 (2005).

3 Zheng, S. Q. *et al.* MotionCor2: anisotropic correction of beam-induced motion for improved cryo-electron microscopy. *Nat Methods* **14**, 331-332, doi:10.1038/nmeth.4193 (2017).

4 Zhang, K. Gctf: Real-time CTF determination and correction. *J Struct Biol* **193**, 1-12, doi:10.1016/j.jsb.2015.11.003 (2016).

5 Zivanov, J. *et al.* New tools for automated high-resolution cryo-EM structure determination in RELION-3. *Elife* **7**, doi:10.7554/eLife.42166 (2018).

6 Punjani, A., Rubinstein, J. L., Fleet, D. J. & Brubaker, M. A. cryoSPARC: algorithms for rapid unsupervised cryo-EM structure determination. *Nature methods* **14**, 290-296, doi:10.1038/nmeth.4169 (2017).

7 Sanchez-Garcia, R. *et al.* DeepEMhancer: a deep learning solution for cryo-EM volume post-processing. *Commun Biol* **4**, 874, doi:10.1038/s42003-021-02399-1 (2021).

8 Yang, Z. *et al.* Structural basis of ligand binding modes at the neuropeptide Y Y1 receptor. *Nature* **556**, 520-524, doi:10.1038/s41586-018-0046-x (2018).

9 Tang, T. *et al.* Structural basis for ligand recognition of the neuropeptide Y Y2 receptor. *Nat Commun* **12**, 737, doi:10.1038/s41467-021-21030-9 (2021).

10 Zhao, C. *et al.* Structural insights into sphingosine-1-phosphate recognition and ligand selectivity of S1PR3-Gi signaling complexes. *Cell Res* **32**, 218-221, doi:10.1038/s41422-021-00567-w (2022).

11 Pettersen, E. F. *et al.* UCSF Chimera--a visualization system for exploratory research and analysis. *J Comput Chem* **25**, 1605-1612, doi:10.1002/jcc.20084 (2004).

12 Emsley, P. & Cowtan, K. Coot: model-building tools for molecular graphics. *Acta Crystallogr D Biol Crystallogr* **60**, 2126-2132, doi:10.1107/s0907444904019158 (2004).

13 Adams, P. D. *et al.* PHENIX: a comprehensive Python-based system for macromolecular structure solution. *Acta Crystallogr D Biol Crystallogr* **66**, 213-221,

doi:10.1107/S0907444909052925 (2010).

14 Williams, C. J. *et al.* MolProbity: More and better reference data for improved all-atom structure validation. *Protein Sci* **27**, 293-315, doi:10.1002/pro.3330 (2018).

15 Pettersen, E. F. *et al.* UCSF ChimeraX: Structure visualization for researchers, educators, and developers. *Protein science : a publication of the Protein Society* **30**, 70-82, doi:10.1002/pro.3943 (2021).

Supplementary Figures

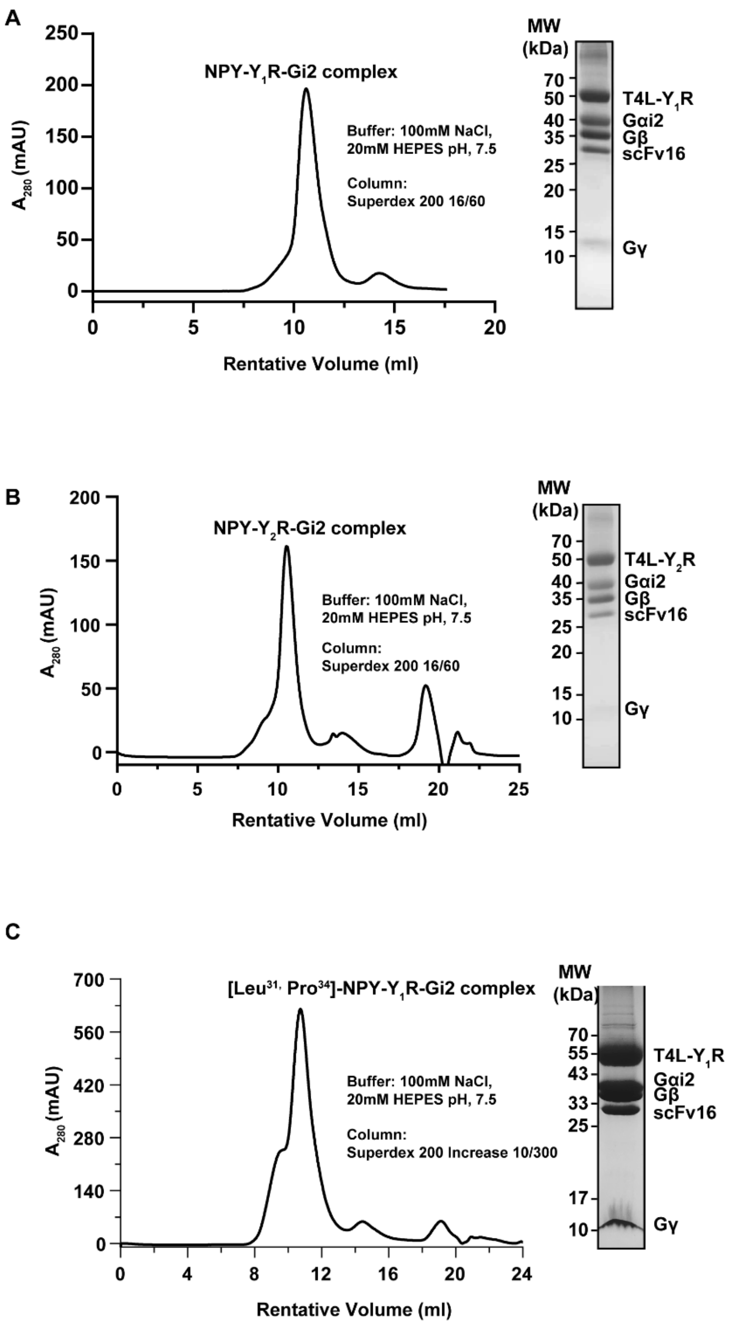

Figure S1. NPYRs bound to NPY or [Leu^{31}, Pro^{34}]-NPY purification.

A. Size-exclusion chromatography elution profiles of the purified NPY-Y_1R-Gi2-scFv16 complex (left) and SDS-PAGE analysis of NPY-Y_1R-Gi2-scFv16 complex. Samples are prepared and repeated over three times.

B. Size-exclusion chromatography elution profiles of the purified NPY-Y_2R-Gi2-scFv16 complex (left) and SDS-PAGE analysis of NPY-Y_2R-Gi2-scFv16 complex. Samples are prepared and repeated over three times.

C. Size-exclusion chromatography elution profiles of the purified [Leu^{31}, Pro^{34}]-NPY-Y_1R-Gi2-scFv16 complex (left) and SDS-PAGE analysis of [Leu^{31}, Pro^{34}]-NPY-Y_1R-

Gi2-scFv16 complex. Samples are prepared and repeated over three times.

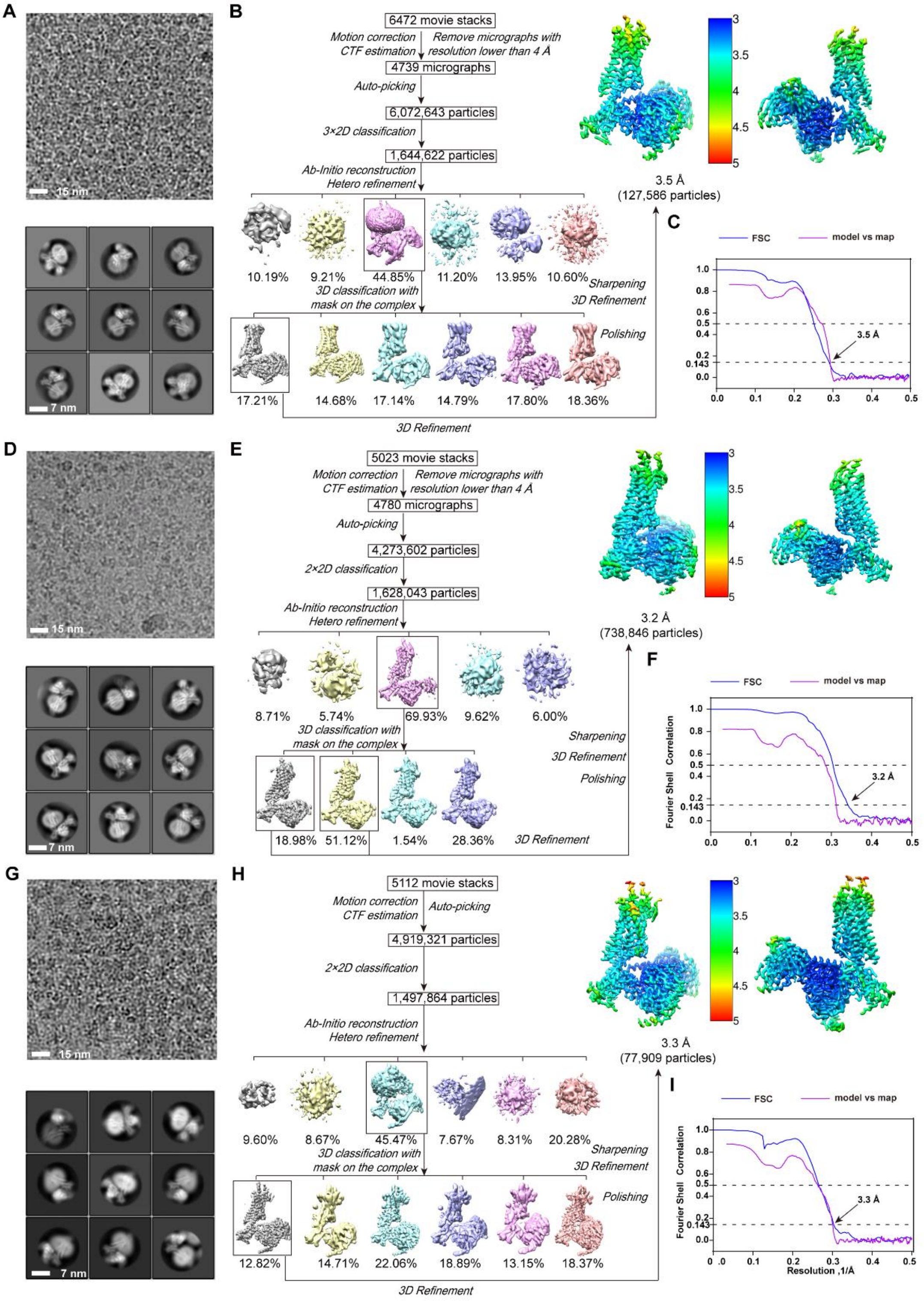

Figure S2. Cryo-EM data collection and cryo-EM map quality.

A. Representative cryo-EM image micrographs of NPY-Y_1R-Gi2 complex (upper panel) and 2D class averages (lower panel), from one of the total 6472 movies.

B. Flow chart of cryo-EM data analysis for the densities of NPY-Y_1R-Gi2 complex. The final resolution of the density is 3.5 Å.

C. The fourier shell correlation (FSC) curve of globally refined NPY–Y_1R–Gi2

complex.
D. Representative cryo-EM image micrographs of NPY-Y_2R-Gi2 complex (upper panel) and 2D class averages (lower panel), from one of the total 5023 movies.
E. Flow chart of cryo-EM data analysis for the densities of NPY-Y_2R-Gi2 complex. The final resolution of the density is 3.2 Å.
F. The fourier shell correlation (FSC) curve of globally refined NPY–Y_2R–Gi2 complex.
G. Representative cryo-EM image micrographs of [Leu^{31}, Pro^{34}]-NPY-Y_1R-Gi2 complex (upper panel) and 2D class averages (lower panel), from one of the total 5112 movies.
H. Flow chart of cryo-EM data analysis for the densities of [Leu^{31}, Pro^{34}]-NPY-Y_1R-Gi2 complex. The final resolution of the density is 3.3 Å.
I. The fourier shell correlation (FSC) curve of globally refined [Leu^{31}, Pro^{34}]-NPY-Y_1R-Gi2 complex.

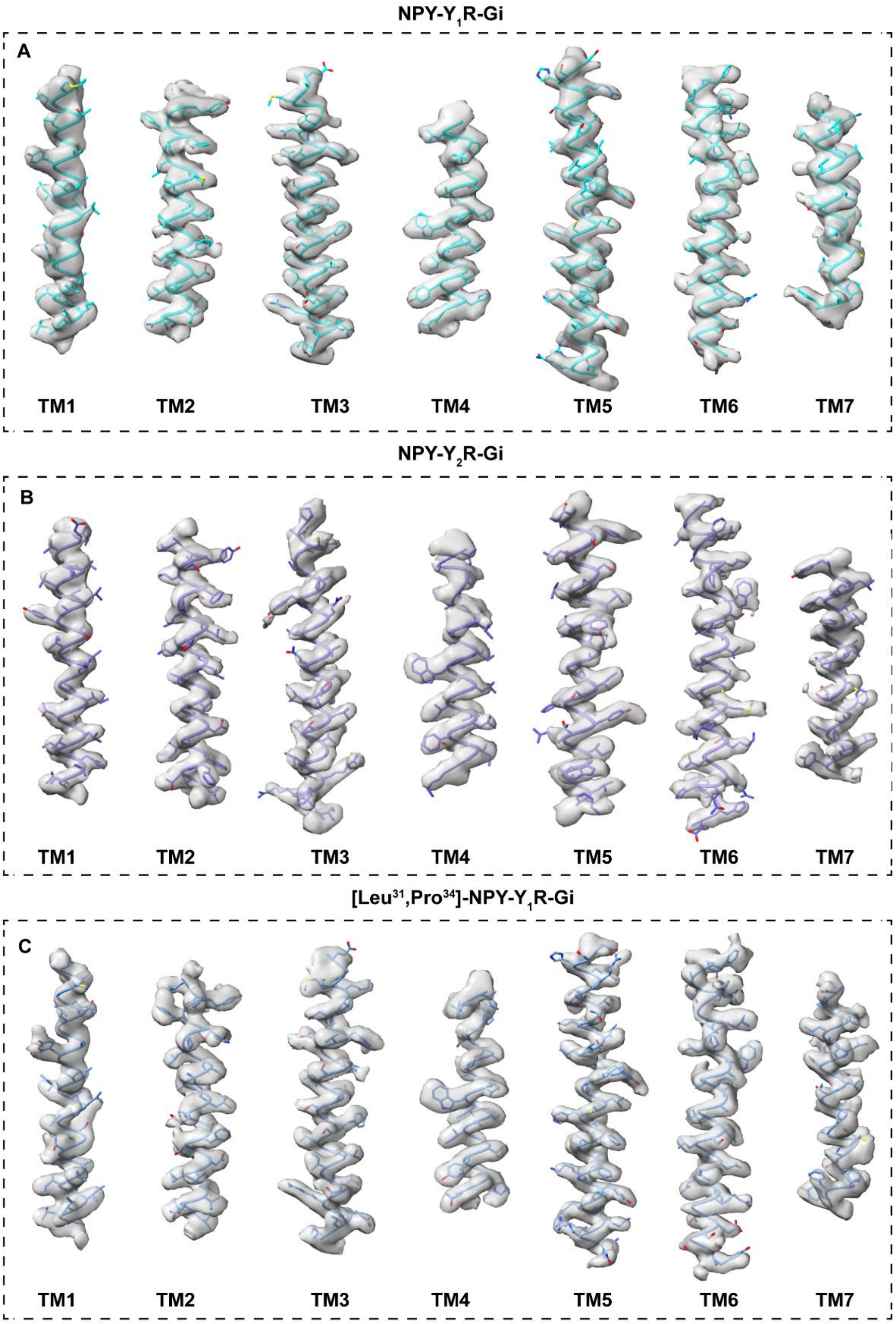

Figure S3. Cryo-EM density representation.

A. Cryo-EM density map and model are shown for seven transmembrane helices of NPY-Y_1R-Gi2 complex.

B. Cryo-EM density map and model are shown for seven transmembrane helices of NPY-Y_2R-Gi2 complex.

C. Cryo-EM density map and model are shown for seven transmembrane helices of

[Leu^{31}, Pro^{34}]-NPY-Y_1R-Gi2 complex.

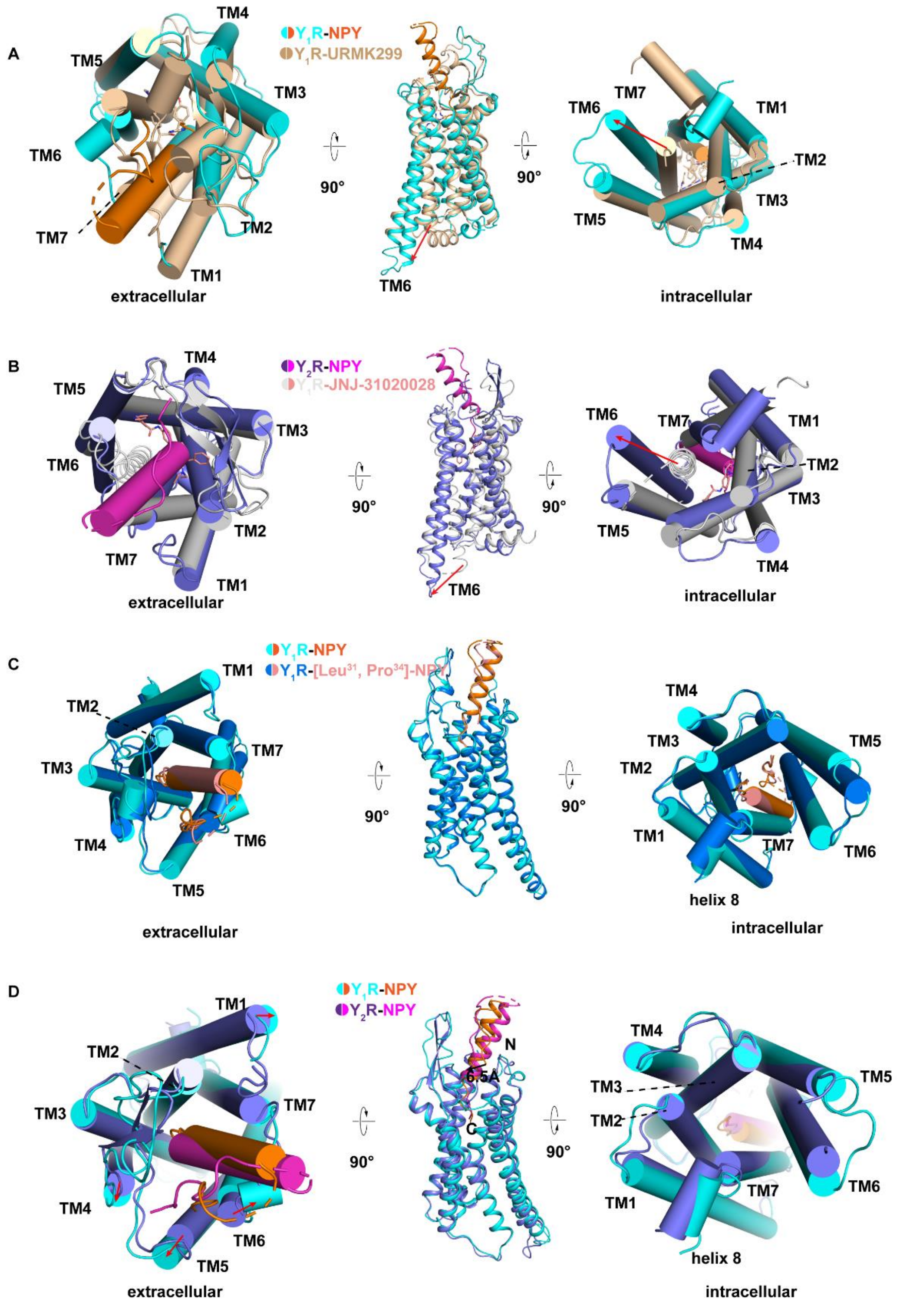

Figure S4.

A. Superimposition of the active structure of NPY-Y$_1$R (cyan and orange) and inactive structure of antagonist URMK299 in complex with Y$_1$R (tan). The extracellular view and intracellular view show the NPY-induced movement of the TM helices. Red arrows indicate the movement of helices.

B. Superimposition of the active structure of NPY-Y$_2$R (medium slate and magenta)

and inactive structure of antagonist JNJ31020028 in complex with Y_2R (silver and medium salmon). The extracellular view and intracellular view show the NPY-induced movement of the TM helices. Red arrows indicate the movement of helices.

C. Superimposition of NPY-Y_1R (cyan and orange) and [Leu^{31}, Pro^{34}]-NPY-Y_1R (medium blue and light salmon). The extracellular view and intracellular view show the movement of the TM helices.

D. Superimposition of NPY-Y_1R (cyan and orange) and NPY-Y_2R (medium slate and magenta). The extracellular view and intracellular view show the movement of the TM helices.

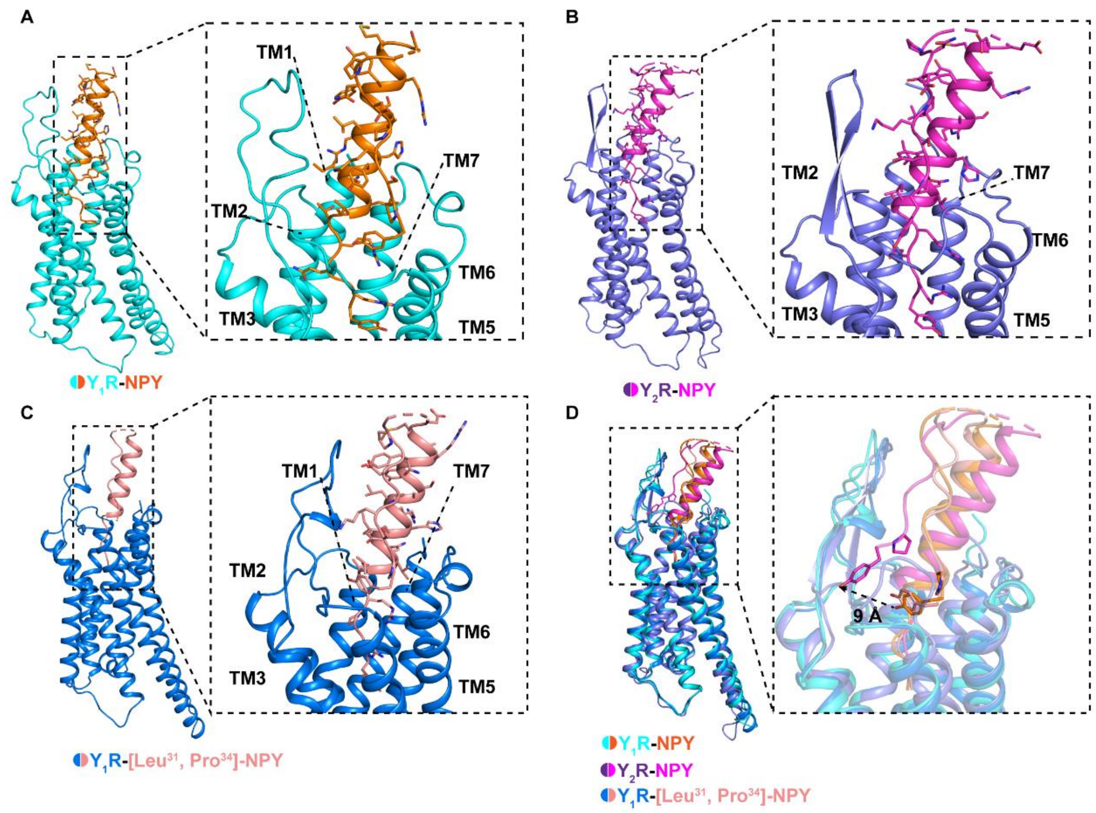

Figure S5.

A. Structure of the Y_1R–NPY complex. The structure of Y_1R (cyan) is shown in cartoon representation and the structure of NPY (orange) is shown in cartoon and sticks representation. The binding cavities for the NPY and Y_1R are highlighted by the dashed box and are shown in detail on the right.

B. Structure of the Y_2R–NPY complex. The structure of Y_2R (medium slate blue) is shown in cartoon representation and the structure of NPY (magenta) is shown in cartoon and sticks representation. The binding cavities for the NPY and Y_2R are highlighted by the dashed box and are shown in detail on the right.

C. Structure of the Y_1R- [Leu^{31}, Pro^{34}]-NPY complex. The structure of Y_1R (medium blue) is shown in cartoon representation and the structure of NPY (light salmon) is shown in cartoon and sticks representation. The binding cavities for the [Leu^{31}, Pro^{34}]-NPY and Y_1R are highlighted by the dashed box and are shown in detail on the right.

D. Comparison of NPY-Y_1R, NPY-Y_2R and [Leu^{31}, Pro^{34}]-NPY-Y_1R. The binding cavities are highlighted by the dashed box and are shown in detail on the right.

Figure S6

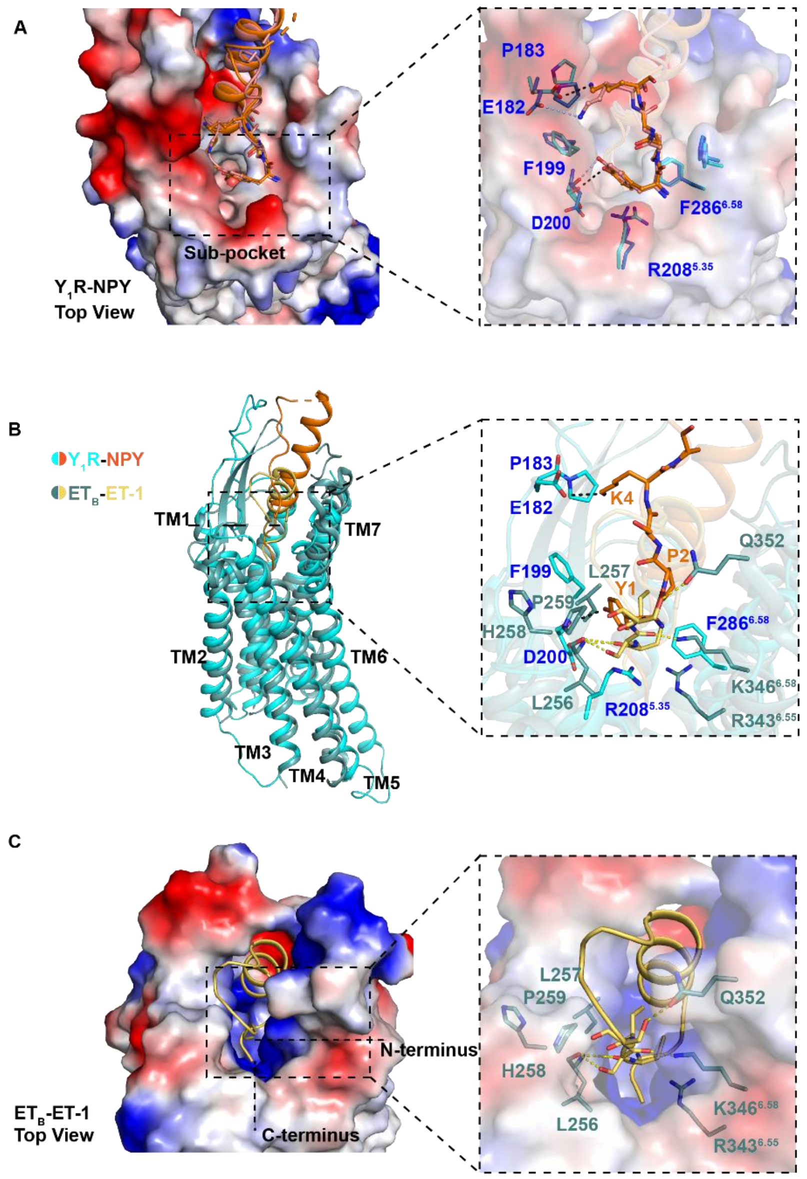

Figure S6.

A. Structural superposition of [Leu^{31}, Pro^{34}]-NPY-Y$_1$R and NPY-Y$_1$R (Y$_1$R is shown as electrostatic surface potential map, [Leu^{31}, Pro^{34}]-NPY bound Y$_1$R and NPY bound Y$_1$R are shown as sticks). Red and blue colors denote negative and positive surface charge, respectively. The details are highlighted by the dashed box and is shown in detail on the right.

B. Structural superposition of NPY-Y$_1$R and ET$_B$-ET-1. The details interactions are highlighted by the dashed box and is shown in detail on the right.

C. Electrostatic surface potential map of ET$_B$ in complex with ET-1 (top view). Red and blue colors denote negative and positive surface charge, respectively. The interactions between N-terminus of ET-1 and ET$_B$ are highlighted by the dashed box and is shown in detail on the right.

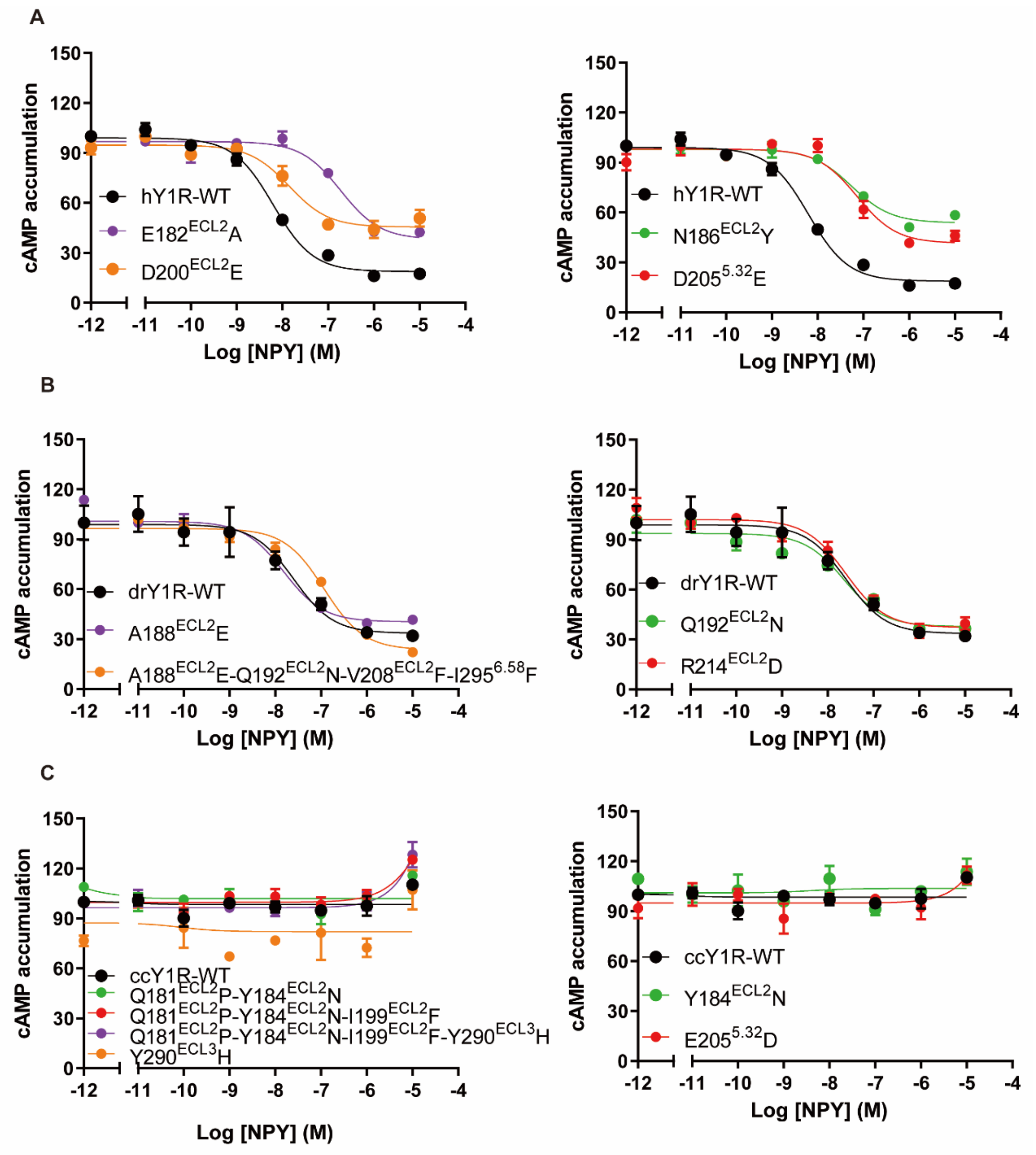

Figure S7. A-C. Representative of effects of hY1R mutants (A), drY1R mutants (B) and ccY1R mutants (C) of the Y_1R on NPY in cAMP accumulation assays. Data represent mean ± s.e.m from three independent experiments performed in triplicate.

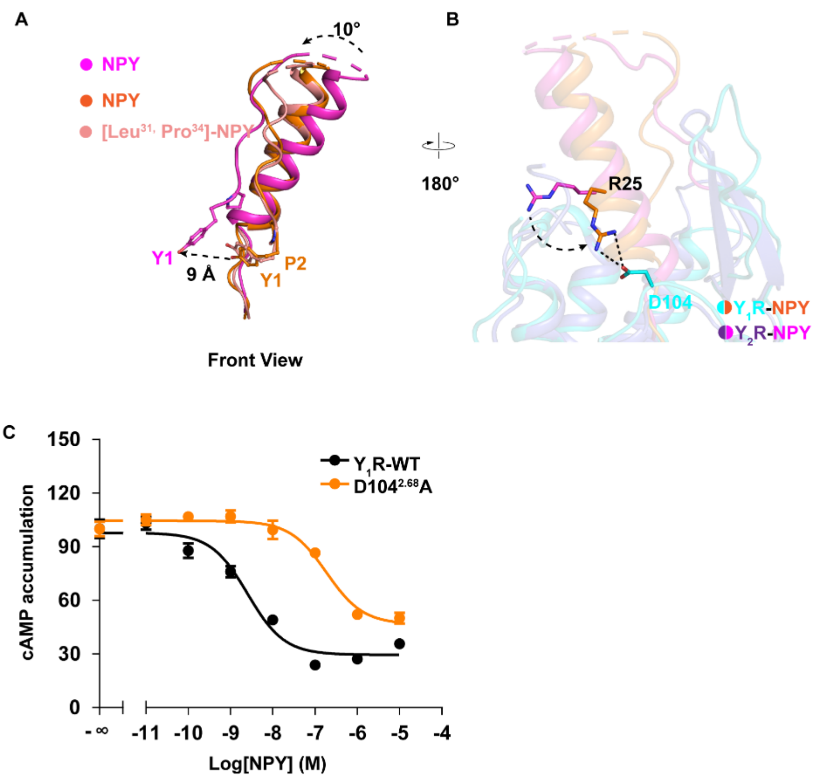

Figure S8.

A. Structural superposition of [Leu^{31}, Pro^{34}]-NPY, NPY in Y_1R and NPY in Y_2R. The rotation of α-helix is shown as black arrows.

B. Comparison of α-helix of NPY binding modes between Y_2R and Y_1R.

C. Concentration–response curves of Y_1R and D104$^{2.68}$A mutation in response to stimulation with NPY, respectively. Values are shown as the mean ± s.e.m. of three experiments (n = 3) performed in triplicate.

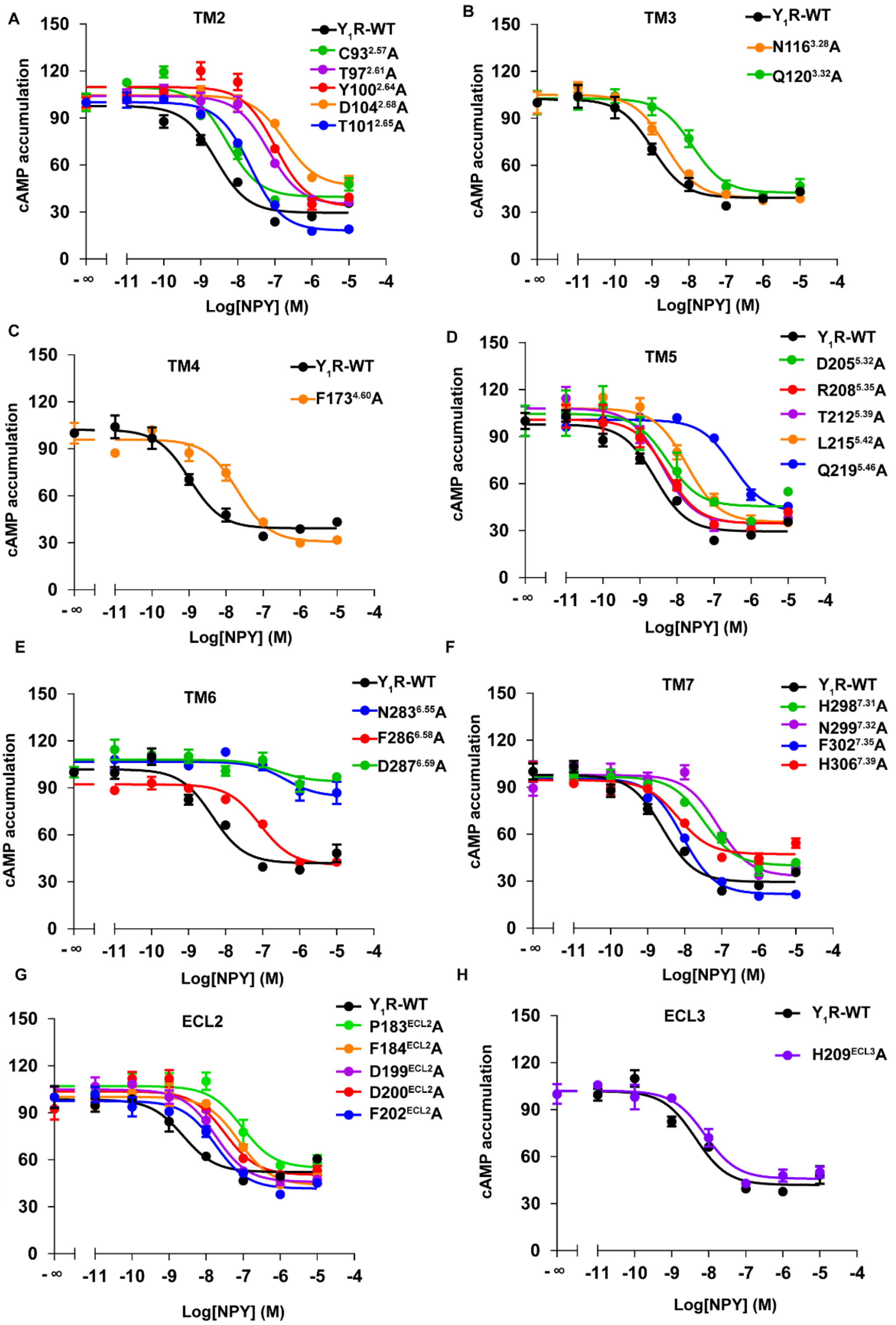

Figure S9. Functional assays of Y$_1$R and mutations by NPY.

A-H. Representative of effects of TM2, TM3, TM4, TM5, TM6, TM7, ECL2 and ECL3 residues of the Y$_1$R on endogenous agonist NPY in cAMP accumulation assays. Data represent mean ± s.e.m from three independent experiments performed in triplicate.

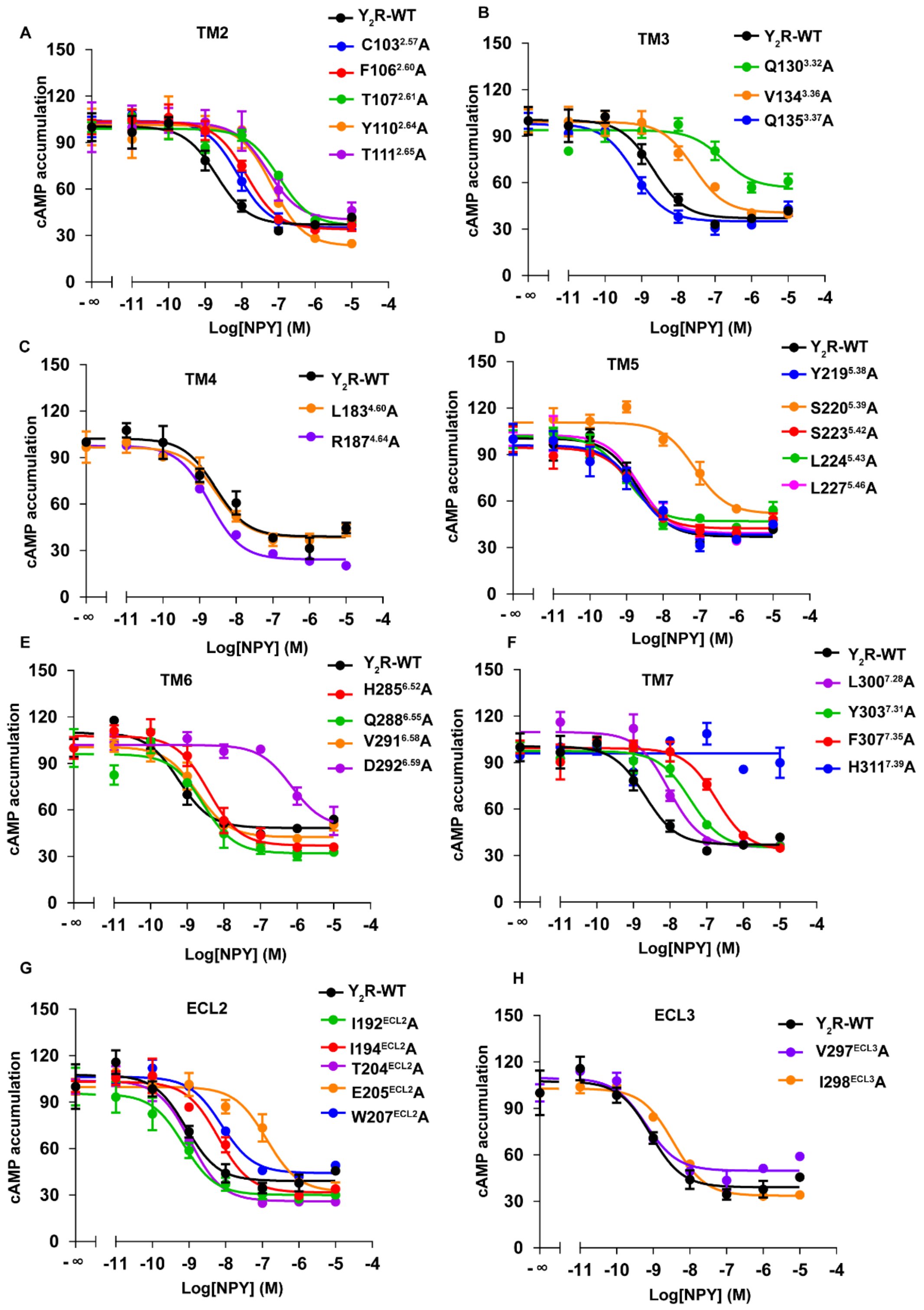

Figure S10. Functional assays of Y_2R and mutations by NPY.

A-H. Representative of effects of TM2, TM3, TM4, TM5, TM6, TM7, ECL2 and ECL3 residues of the Y_2R on endogenous agonist NPY in cAMP accumulation assays. Data represent mean ± s.e.m from three independent experiments performed in triplicate.

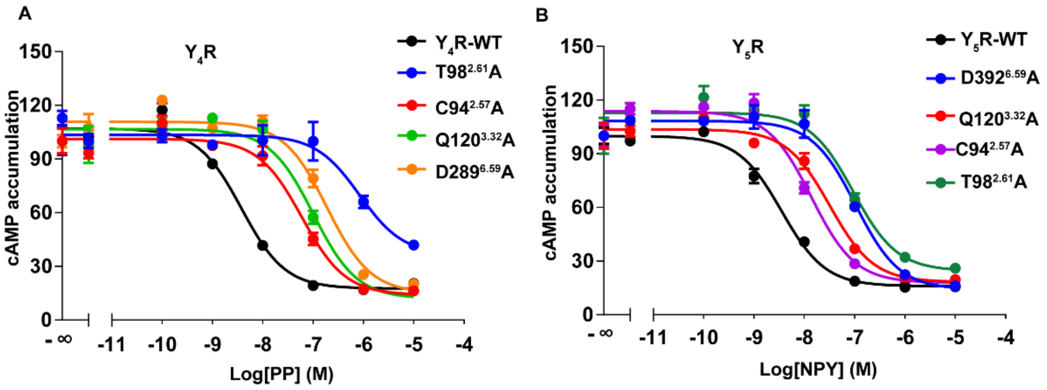

Figure S11. Functional assays of Y_4R, Y_5R and mutations by PP, NPY respectively. A-B. Representative of effects of identified common sites of the Y_4R and Y_5R on PP and NPY in cAMP accumulation assays. Data represent mean ± s.e.m from three independent experiments performed in triplicate.

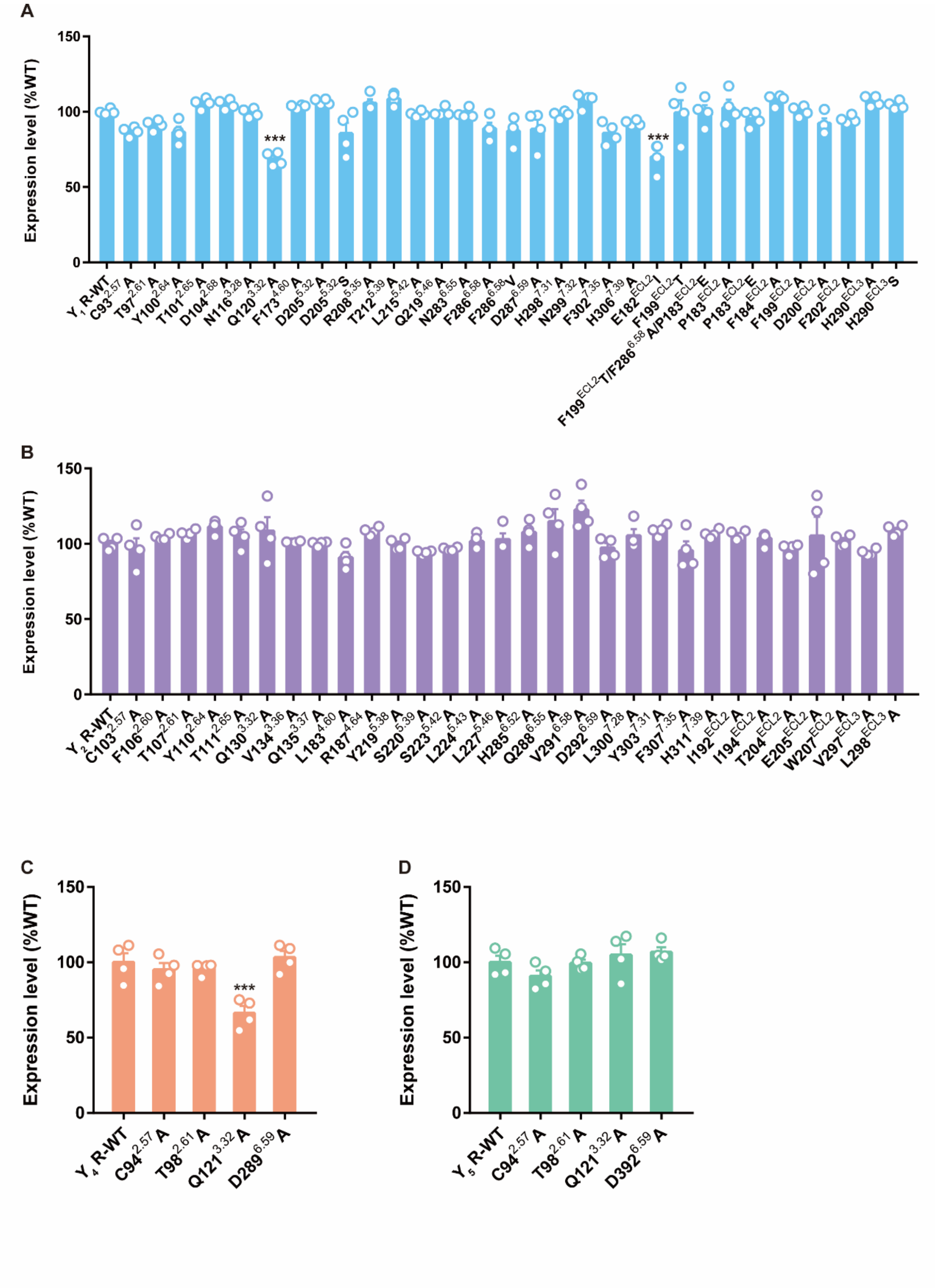

Figure S12. A-D. Expression level of wild-type and mutants of Y_1R, Y_2R, Y_4R and Y_5R. Data are presented as the mean ± s.e.m of three independent experiments performed in triplicate. Statistical differences between WT and mutants were determined by one-way of variance ANOVA with Dunnett's test. **P* < 0.05; ***P*< 0.01; ****P* < 0.001; ns, no significant difference.

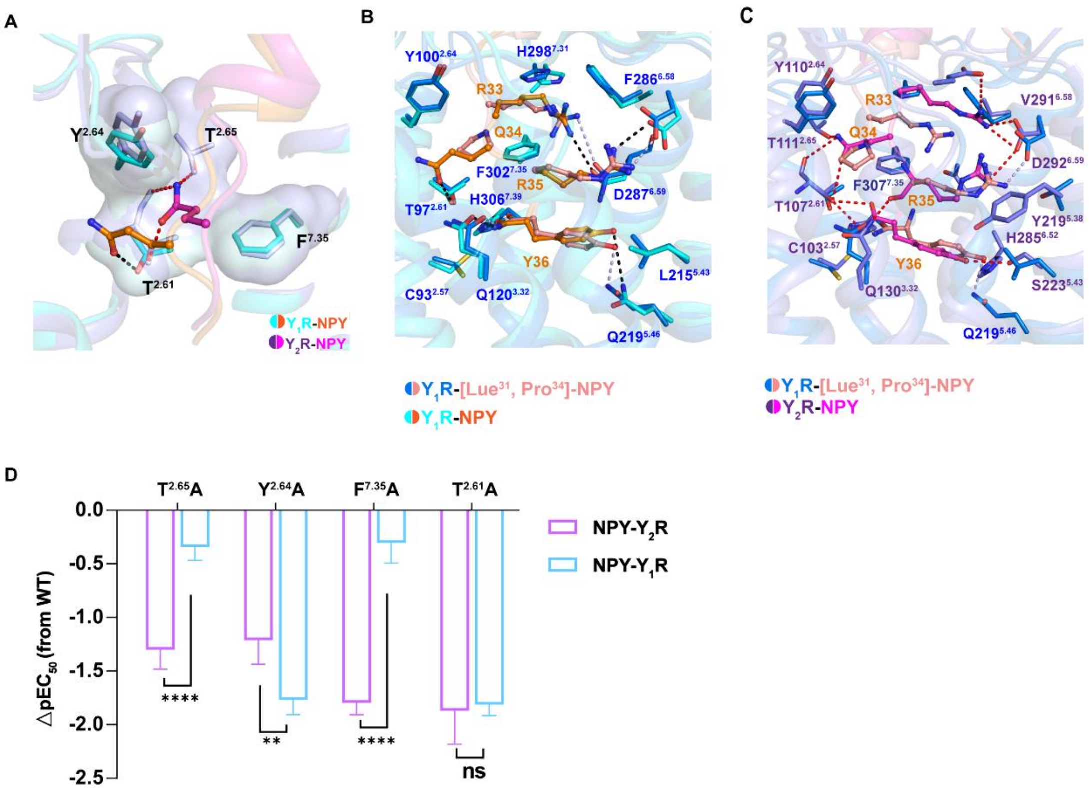

Figure S13. Comparison interface of peptide agonist-bound Y_1R and Y_2R structures.

A. Comparison of the C-terminus of NPY bound Y_1R and NPY bound Y_2R.

B. Comparison of the C-terminus of NPY bound Y_1R and [Leu^{31}, Pro^{34}]-NPY bound Y_1R.

C. Comparison of the C-terminus of NPY bound Y_2R and [Leu^{31}, Pro^{34}]-NPY bound Y_1R.

D. NPY-induced cAMP accumulation assays of the residues interacting with Q^{34} in Y_1R and Y_2R. Bars represent differences in calculated NPY potency [pEC_{50}] for each mutant relative to the wild-type receptor (WT). *P* values were determined by Unpaired t test. **, $P < 0.01$; ****, $P \leq 0.0001$; ns, no significant difference.

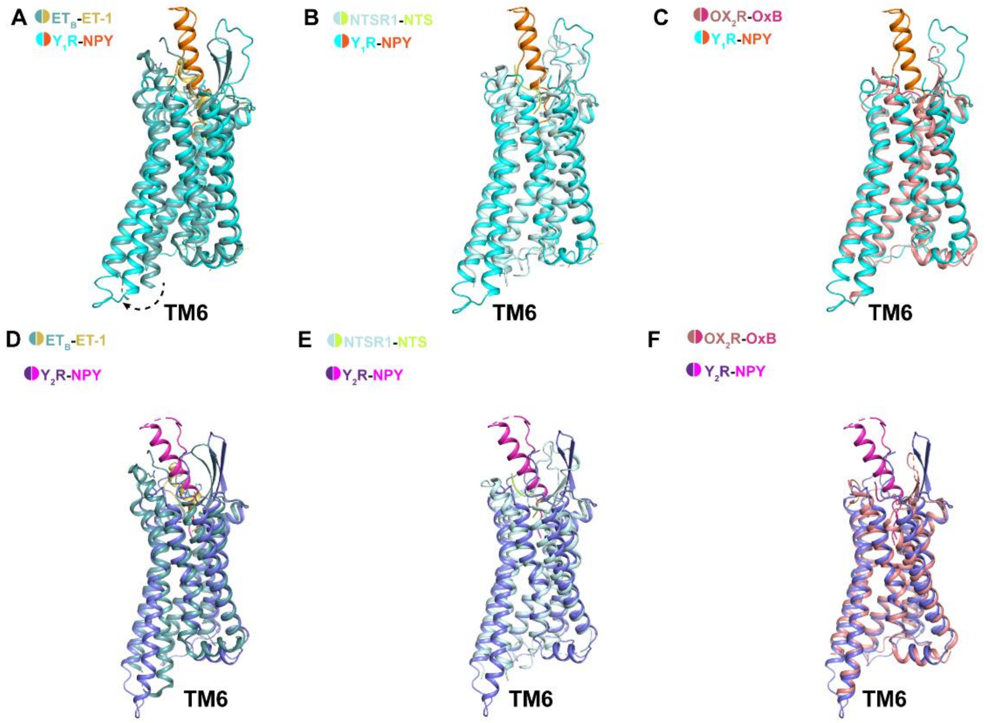

Figure S14. Comparison of NPY-Y_1R or NPY-Y_2R with active structures of other receptors in complex with peptide agonist.

A-C. Structural comparison of NPY-Y_1R with ET_B-ET-1(A), NTSR1-NTS (B) and Ox2r-OxB(C). D-F. Structural comparison of NPY-Y_2R with ET_B-ET-1(D), NTSR1-NTS (E) and Ox2r-OxB (F).

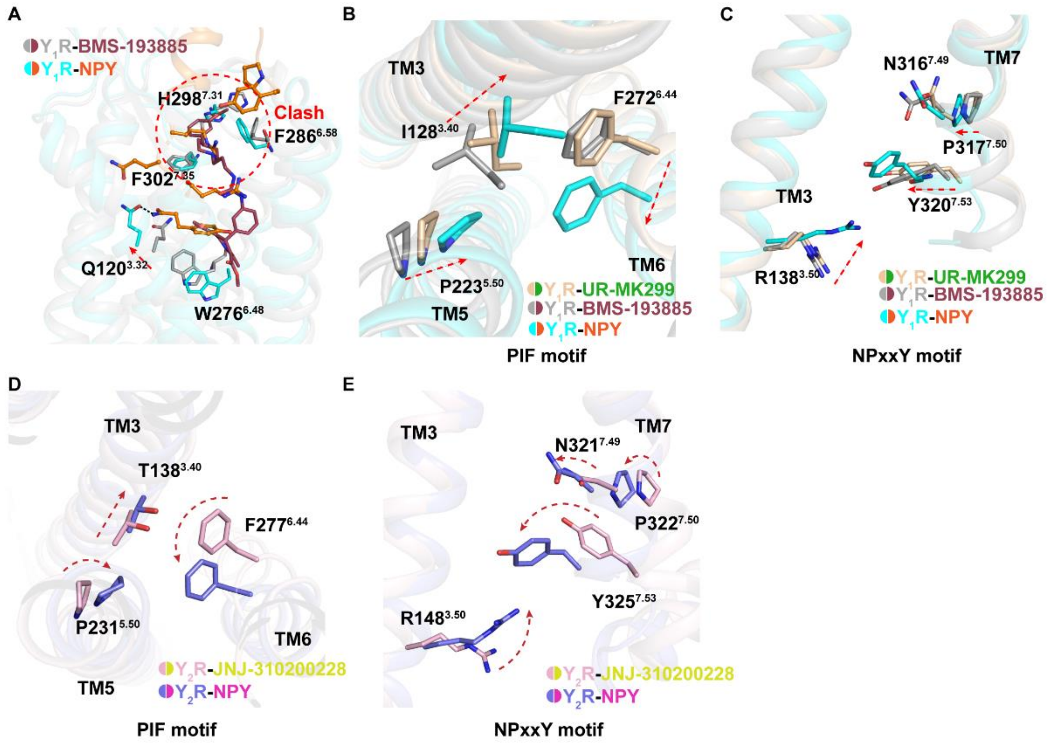

Figure S15. Activation mechanism of Y_1R and Y_2R.

A. Structural superposition of the NPY-Y_1R and antagonist (BMS-194885)-bound Y_1R.

B-C. Conformational changes of the conserved "micro-switches" upon Y_1R activation. PIF motif (B) and NPxxY motif (C). The conformational changes of residue side chains are shown as red arrows upon receptor activation.

D-E. Conformational changes of the conserved "micro-switches" upon Y_2R activation. PIF motif (D) and NPxxY motif (E). The conformational changes of residue side chains are shown as red arrows upon receptor activation.

Supplementary Tables

Supplementary Table S1 Cryo-EM data collection, refinement and validation statistics.

Parameters	NPY-Y_1R Gi2 complex	NPY-Y_2R - Gi2 complex	[Leu31, Pro34]-NPY-Y_1R- Gi2 complex
**Data collection and processing**			
Magnification	130,000	165,000	165,000
Voltage (kV)	300	300	300
Electron exposure (e–/Å^2)	66	61	64
Defocus range (μm)	-1 to -1.8	-1 to -1.8	-1 to -1.8
Pixel size (Å)	0.46	0.85	0.85
Symmetry imposed	C1	C1	C1
Initial particle images (no.)	6,072,643	4,273,602	4,919,321
Final particle images (no.)	127,586	738,846	77,909
Map resolution (Å)	3.5	3.2	3.3
FSC threshold	0.143	0.143	0.143
Map resolution range (Å)	3.0-5.0	3.0-5.0	3.0-5.0
**Refinement**			
Initial model used (PDB code)			
Model resolution (Å)	3.7	3.4	3.5
FSC threshold	0.5	0.5	0.5
Model composition			
Non-hydrogen atoms	9318	9292	9333
Protein residues	1181	1186	1183
B factors (Å)			
Protein	155.16	171.85	93.01
R.m.s. deviations			
Bond lengths (Å)	0.006	0.004	0.003
Bond angles (°)	1.029	0.671	0.597
Validation			
MolProbity score	1.71	1.96	1.35
Clashscore	6.64	11.55	6.41
Poor rotamers (%)	0.10	0.10	0.20
Ramachandran plot			
Favored (%)	95.01	94.35	98.20
Allowed (%)	3.91	5.65	1.80
Disallowed (%)	0.00	0.00	0.00

Supplementary Table 2. Summary of NPY-induced cAMP inhibition assay of NPYRs. Related to the Methods section “Cyclic adenosine 3′,5′-monophosphate (cAMP) assay.

**Receptors**	**EC$_{50}$ (nM)**	**Fold**	**Emax (%WT)**	**n**	**Expression level (%WT)**
**NPYRs**			**NPY**		
**NPY$_1$R-WT**	2.53±3.33	1.00	100	3	100
T101$^{2.65}$A	21.82±1.15	8.62	120.07±2.05	3	105.50±1.79
Y100$^{2.64}$A	103.20±1.38	40.79	111.79±4.99	3	86.40±3.61
C93$^{2.57}$A	5.11±1.41	2.02	102.59±4.24	3	86.86±1.59
T97$^{2.61}$A	66.95±1.26	26.46	101.39±3.15	3	91.22±1.77
D104$^{2.68}$A	186.10±1.33	73.56	84.85±3.39	3	104.90±1.62
N116$^{3.28}$A	2.40±1.27	0.95	104.52±2.72	3	99.26±1.51
Q120$^{3.32}$A	12.27±1.44	4.85	95.48±4.05	3	68.65±2.24
F173$^{4.60}$A	20.47±1.36	8.09	103.47±3.61	3	103.50±0.60
R208$^{5.35}$A	5.27±1.44	2.08	97.23±4.22	3	105.70±2.66
D205$^{5.32}$A	5.41±2.00	2.14	86.52±7.24	3	106.70±0.95
D205$^{5.32}$S	5.19±1.34	2.05	104.90±5.40	3	85.61±6.90
T212$^{5.39}$A	3.98±1.39	1.57	107.50±4.11	3	108.40±2.08
L215$^{5.42}$A	18.63±1.32	7.36	106.12±3.73	3	98.60±0.93
Q219$^{5.46}$A	319.10±1.3	126.13	86.32±3.23	3	99.73±1.5
F286$^{6.58}$A	85.22±1.31	33.68	85.91±2.79	3	88.97±3.75
F286$^{6.58}$V	56.61±1.27	22.38	113.7±5.07	3	87.18±4.3
D287$^{6.59}$A	233.90±5.76	92.45	23.10±4.91	3	88.47±6.21
N283$^{6.55}$A	395.1±3.02	156.17	37.24±5.24	3	98.86±1.59
H306$^{7.39}$A	6.48±1.46	2.56	69.22±3.22	3	92.64±0.86
H298$^{7.31}$A	32.28±1.35	12.76	82.99±3.03	3	98.13±1.22
N299$^{7.32}$A	78.47±1.39	31.02	94.55±4.23	3	107.5±2.42
F302$^{7.35}$A	8.61±1.22	3.40	108.37±2.71	3	85.68±3.53
F202^{ECL2}A	17.81±1.34	7.04	120.85±2.99	3	94.88±1.26
F199^{ECL2}A	17.89±1.36	7.07	127.26±3.31	3	100.40±1.74
F199^{ECL2}T	12.02±1.21	4.75	109.70±3.81	3	99.25±8.39
F184^{ECL2}A	75.83±1.34	29.97	121.62±3.23	3	108.00±1.82

D200^{ECL2}A	31.8±1.56	12.57	115.29±4.26	3	92.32±3.28
E182I	5.22±1.14	2.06	107.9±2.5	3	70.08±4.8
P183^{ECL2}A	92.65±1.59	36.62	112.88±4.87	3	102.60±5.38
P183^{ECL2}E	36.01±1.35	14.23	113.9±6.24	3	95.32±2.62
F199^{ECL2}T/F286$^{6.58}$V/P183^{ECL2}E	4967.00±1.57	1963.24	165.00±27.14	3	99.86±4.44
H290^{ECL3}A	7.88±1.44	3.11	93.81±3.73	3	107.10±1.79
H290^{ECL3}S	2.07±1.28	0.82	95.48±4.15	3	104.4±1.36
**NPY$_2$R-WT**	2.05±1.49	1.00	100	3	100
F106$^{2.60}$A	13.55±1.35	6.61	109.65±3.83	3	104.30±1.01
T107$^{2.61}$A	102.90±1.41	50.20	98.63±4.37	3	106.70±1.44
T111$^{2.65}$A	51.30±2.01	25.02	98.88±8.15	3	105.50±4.07
Y110$^{2.64}$A	66.62±1.65	32.50	123.26±7.61	3	111.20±2.19
C103$^{2.57}$A	7.8±1.29	3.80	108.74±3.25	3	97.18±6.45
V134$^{3.36}$A	25.89±1.56	12.59	92.56±4.64	3	101.10±0.29
Q130$^{3.32}$A	160.20±2.05	78.15	58.63±5.46	3	108.40±9.27
Q135$^{3.37}$A	0.64±1.36	0.31	99.15±3.56	3	100.20±0.79
L183$^{4.60}$A	2.8±1.56	1.37	91.95±4.40	3	90.87±3.65
R187$^{4.64}$A	1.88±1.15	0.92	116.04±1.84	3	108.4±1.34
Y219$^{5.38}$A	1.92±1.69	0.94	90.85±5.29	3	99.67±1.86
S223$^{5.42}$A	1.71±1.56	0.83	82.17±4.11	3	96.48±0.6
L224$^{5.43}$A	0.97±1.47	0.47	86.74±3.91	3	101.60±2.44
L227$^{5.46}$A	2.16±1.42	1.05	99.91±3.85	3	102.80±4.03
S220$^{5.39}$A	72.06±1.53	35.15	92.72±4.86	3	94.22±0.63
H285$^{6.52}$A	3.74±1.46	1.82	114.9±4.66	3	107.6±3.87
Q288$^{6.55}$A	2.68±1.67	1.31	104.26±5.6	3	114.70±8.39
V291$^{6.58}$A	1.83±1.41	0.89	94.71±3.55	3	122.4±6.24
D292$^{6.59}$A	697.00±1.61	340.00	86.47±6.07	3	97.18±3.22
F307$^{7.35}$A	181.9±1.45	88.73	103.97±5.00	3	95.44±6.19
Y303$^{7.31}$A	35.57±1.37	17.35	98.41±3.46	3	108.90±1.79
L300$^{7.28}$A	9.24±1.38	4.51	117.05±4.53	3	105.30±4.39
H311$^{7.39}$A	ND	ND	ND	3	106.50±1.39
I194^{ECL2}A	6.25±1.36	3.05	104.35±3.93	3	103.50±2.24
I192^{ECL2}A	0.71±1.57	0.35	95.38±5.38	3	105.80±1.45

T204^{ECL2}A	1.07±1.24	0.52	113.80±3.15	3	96.90±1.72
E205^{ECL2}A	131.60±1.46	63.90	98.79±5.21	3	105.20±12.72
W207^{ECL2}A	7.72±1.28	3.77	90.78±2.85	3	101.90±1.92
V297^{ECL3}A	0.63±1.48	0.31	87.51±4.26	3	94.22±1.12
L298^{ECL3}A	3.53±1.18	1.72	101.07±1.98	3	108.80±1.75
**NPY$_4$R-WT**	3.78±1.28	1.00	100	3	100
T98$^{2.61}$A	833.2±1.50	220.42	74.84±6.77	3	95.88±2.02
C94$^{2.57}$A	59.55±1.26	15.75	97.56±3.9	3	95.12±4.48
Q121$^{3.32}$A	100.50±1.3	26.46	105.94±5.03	3	66.21±4.8
D289$^{6.59}$A	189.10±1.32	50.03	107.10±5.34	3	103.2±4.45
**NPY$_5$R-WT**	3.54±1.16	1.00	100	3	100
D392$^{6.59}$A	104.80±1.24	29.60	112.26±4.04	3	106.70±3.21
Q121$^{3.32}$A	32.09±1.24	9.06	101.71±3.23	3	104.80±7.09
C94$^{2.57}$A	13.8±1.26	3.90	114.24±4.05	3	90.64±4.04
T98$^{2.61}$A	94.24±1.37	26.62	105.47±5.56	3	99.33±2.35

ND means no detectable due to the low signal.

Supplementary Table 3. Summary of NPY-induced cAMP inhibition assay of six classics vertebrate NPYRs. Related to the Methods section “Cyclic adenosine 3′,5′-monophosphate (cAMP) assay.

Specie	EC_{50} (nM)	Emax (% $hNPY_1R$-WT)	n
Homo Sapiens	6.27±0.91	100	3
Chrysemys picta bellii	1.13±0.22	87.51±2.54	3
Danio rerio	25.00±13.71	81.37±6.34	3
Petromyzon marinus	37.80±9.68	88.58±3.26	3
Scyliorhinus canicula	175.10±99.65	59.21±5.50	3
Carcharodon carcharias	ND	ND	3

ND means no detectable due to the low signal.

Supplementary Table 4. Summary of NPY-induced cAMP inhibition assay of hY_1R, drY_1R and ccY_1R. Related to the Methods section "Cyclic adenosine 3′,5′-monophosphate (cAMP) assay.

Receptor	EC_{50} (nM)	Emax (%WT)	n	Expression level (%WT)
**NPY_1Rs**		**NPY**		
**hY_1R**	6.27±0.91	100	3	100
$F199^{ECL2}I$	826.70±113.40	114.20±3.92	3	111.54±3.76
$F199^{ECL2}V$-$F286^{6.58}I$	ND	ND	3	86.70±4.26
$E182^{ECL2}A$	186.90±54.70	73.18±3.49	3	96.83±7.89
$D200^{ECL2}E$	14.02±5.18	61.34±3.34	3	98.81±4.66
$N186^{ECL2}Y$	54.65±16.32	55.01±2.48	3	98.21±4.50
$D205^{5.32}E$	72.05±27.37	70.46±4.21	3	98.60±2.33
**drY_1R**	25.00±13.71	100	3	100
$V208^{ECL2}F$	3796.00±2327.00	134.50±26.92	3	95.54±6.41
$V208^{ECL2}F$-$I295^{6.58}F$	2068.00±292.10	120.70±4.83	3	104.38±7.68
$A188^{ECL2}E$	15.10±3.24	92.39±2.42	3	85.11±4.42
$A188^{ECL2}E$-$Q192^{ECL2}N$-$V208^{ECL2}F$-$I295^{6.58}F$	117.00±26.30	111.55±3.56	3	102.67±3.94
$Q192^{ECL2}N$	22.84±9.32	85.62±4.05	3	96.52±10.22
$R214^{ECL2}D$	24.65±7.93	99.13±3.69	3	95.69±8.57
**ccY_1R**	ND	ND	3	100
$I199^{ECL2}F$	ND	ND	3	98.96±4.14
$Q181^{ECL2}P$-$Y184^{ECL2}N$-$I199^{ECL2}F$-$Y290^{ECL3}H$	ND	ND	3	92.64±4.17
$Q181^{ECL2}P$-$Y184^{ECL2}N$	ND	ND	3	97.02±0.76
$Q181^{ECL2}P$-$Y184^{ECL2}N$-$I199^{ECL2}F$	ND	ND	3	103.56±3.26
$Y290^{ECL3}H$	ND	ND	3	98.38±5.03
$Y184^{ECL2}N$	ND	ND	3	105.39±5.77
$E205^{5.32}D$	ND	ND	3	102.41±9.81

ND means no detectable due to the low signal.
